# Novel insights into the pathogenesis of thyroid eye disease through ferroptosis-related gene signature and immune infiltration analysis

**DOI:** 10.18632/aging.205685

**Published:** 2024-03-25

**Authors:** Yunyan Ye, Lei Dai, Joseph Mugaanyi, Weina Fu, Feng Hu

**Affiliations:** 1 Department of Ophthalmology, Ningbo Medical Centre Lihuili Hospital, Ningbo University, Ningbo 315040, Zhejiang, China; 2 Department of Hepato-Pancreato-Biliary Surgery, Ningbo Medical Centre Lihuili Hospital, Ningbo University, Ningbo 315040, Zhejiang, China

**Keywords:** thyroid eye disease, ferroptosis, immune infiltration, multiple omics, diagnostic model

## Abstract

Thyroid eye disease (TED) has brought great physical and mental trauma to patients worldwide. Although a few potential signaling pathways have been reported, knowledge of TED remains limited. Our objective is to explore the fundamental mechanism of TED and identify potential therapeutic targets using diverse approaches. To perform a range of bioinformatic analyses, such as identifying differentially expressed genes (DEGs), conducting enrichment analysis, establishing nomograms, analyzing weighted gene correlation network analysis (WGCNA), and studying immune infiltration, the datasets GSE58331, GSE105149, and GSE9340 were integrated. Further validation was conducted using qPCR, western blot, and immunohistochemistry techniques. Eleven ferroptosis-related DEGs derived from the lacrimal gland were originally screened. Their high diagnostic value was proven, and diagnostic prediction nomogram models with high accuracy and robustness were established by using machine learning. A total of 15 hub gene-related DEGs were identified by WGCNA. Through CIBERSORTx, we uncovered five immune cells highly correlated with TED and found several special associations between these immune cells and the above DEGs. Furthermore, EGR2 from the thyroid sample was revealed to be closely negatively correlated with most DEGs from the lacrimal gland. High expression of APOD, COPB2, MYH11, and MYCN, as well as CD4/CD8 T cells and B cells, was verified in the periorbital adipose tissues of TED patients. To summarize, we discovered a new gene signature associated with ferroptosis that has a critical impact on the development of TED and provides valuable insights into immune infiltration. These findings might highlight the new direction and therapeutic strategies of TED.

## INTRODUCTION

Graves’ ophthalmopathy (GO), commonly associated with Graves’ hyperthyroidism (GH), is a retrobulbar autoimmune condition referred to as thyroid eye disease (TED) [1–3]. It was reported that 25%-50% of patients with Graves’ disease (GD) presented varying degrees of ocular symptoms [4]. The typical clinical characteristics include eyelid retraction, ocular dyskinesia, diplopia, exophthalmos, and strabismus. Moreover, dysthyroid optic neuropathy (DON) might develop into visual loss in severe cases [5–7].

TED is recognized as an inflammatory disease with orbital and extraocular muscle involvement. The primary pathogenesis is well known as immune-induced TSH receptor and IGF-1 receptor injury in ocular connective tissues (OCT) [8–10]. Although glucocorticoids [11], surgery [12], radiotherapy [13] and targeted drugs (tocilizumab [14] and teprotumumab [15]) can partially benefit patients, promising therapies remain absent due to the side effects and high cost of current measures.

Most studies have proposed that the positive feedback effect of inflammatory cytokines runs throughout the TED process. Immune cells infiltrating periorbital tissues release inflammatory mediators to activate OFs, which in turn secrete cytokines to promote the homing and infiltration of immune cells [2, 5, 16]. However, current insights into the mechanisms of TED are still unclear. With the rise of high-throughput sequencing technology, potential biomarkers related to TED have emerged. Wescombe et al. [17] revealed that CASQ2 and SDH4 were highly expressed in TED via an autoimmunity trigger mechanism. Zhao et al. [18] found that differentially expressed genes (DEGs) associated with the cell cycle (UBE2C), encoding proteasome (PSMA1), and signal recognition particle (SRP14) could have significant involvement in the development of TED. Further investigation also identified several (e.g., PTX3, CCL2, HOXB2, SERPINA1, HSP90B1, and CANX) as novel biomarkers of TED [19–22]. Although these valuable studies enriched our understanding of the underlying pathological processes in different target tissues of TED, whether there is a regulatory relationship between DEGs in each target tissue remains unknown. Furthermore, the majority of investigations only emphasized DEG screening and pathway speculation without in-depth exploration and verification.

For the first time, we incorporated microarray information from the Gene Expression Omnibus (GEO) repository in our study and discovered the significant involvement of ferroptosis-associated genes in TED. We also employed machine learning to establish a ferroptosis-related diagnostic nomogram and validate its accuracy for TED. Moreover, we integrated the DEGs of the thyroid, lacrimal gland, and periorbital adipose tissue and used correlation analysis to investigate possible relationships. Furthermore, an analysis of immune infiltration was conducted to investigate possible relationships between DEGs and immune cells. All the findings will refresh our understanding of the development of TED and support novel directions for further exploration.

## MATERIALS AND METHODS

### Data acquisition

We focused on two gene expression datasets (GSE105149 and GSE58331) related to TED, utilizing the GEO database’s information retrieval system (https://www.ncbi.nlm.nih.gov/geo/) [23–28]. Both microarray datasets originated from the same platform, GPL570 [HG-U133_Plus_2] Affymetrix Human Genome U133 Plus 2.0 Array, with the organism *Homo sapiens* and an experimental type of expression profiling by array. According to the inclusion criteria for TED and normal lacrimal samples, 4 cases of TED and 7 control cases derived from GSE105149 [23] were included, as well as 8 cases of TED and 7 control cases derived from GSE58331 [24–28]. The raw expression matrix and annotation of the above samples were downloaded and integrated for subsequent bioinformatic analysis. We employed the ComBat function of the SVA package [29] in R (version 4.2.1) [30] to eliminate the batch effects and visualized the data distribution using different diagrams [31].

### Screening of ferroptosis-related DEGs

To investigate whether ferroptosis is involved in the pathogenesis of TED, we first obtained the union set of genes associated with ferroptosis from both the *Homo sapiens* organism and the FerrDb V2 database (http://www.zhounan.org/ferrdb/current/operations/download.html) [32]. Then, ferroptosis-related DEGs (F-DEGs) were screened out via the intersection with the DEG set of TED.

### Expression analysis and diagnostic value assessment

We assessed the differential expression of F-DEGs in 14 normal and 12 TED tissues, with data normalized and log2 transformed. To assess the diagnostic capability of F-DEGs in TED, we used receiver operating characteristic (ROC) curves to perform a series of analyses. To measure the diagnostic value, the area under the curve (AUC) was calculated. An AUC>0.9 was defined as high accuracy, 0.7<AUC≤0.9 was defined as medium accuracy, and 0.5<AUC≤0.7 was defined as low accuracy [33].

### Construction of the F-DEG-related diagnostic model

Based on the expression profiles above, we performed univariate binary logistic regression analysis using the glmnet package [34] in R to further screen variables. Then, the variables were included in the multivariate analysis and the model correlation test if they met the threshold of *p* < 0.01. F-DEGs were chosen for the diagnostic model, and their odds ratio (OR) was visualized by a forest diagram [35]. On this basis, we constructed an F-DEG-related diagnostic nomogram for TED patients. The model was calibrated [36] and evaluated by employing diagnostic ROC and decision curve analysis (DCA) to verify its precision and robustness. The concordance index (C-Index) and Akaike information criterion (AIC) [37, 38] were subsequently computed. Furthermore, we applied LASSO regression [35, 39], which is a supervised machine learning method, to identify the most relevant variables for diagnostic model establishment. Calibration, diagnostic ROC, and DCA were performed to verify the model’s accuracy and reliability.

### Gene enrichment analysis

We conducted Kyoto Encyclopedia of Genes and Genomes (KEGG) pathway analysis, as well as Gene Ontology (GO) analysis, for all DEGs using the “tidyr”, “ggplot2”, and “clusterProfiler” packages in R (version 4.0.3, www.r-project.org) [30]. Bubble diagrams and circle plots were applied for visualization.

### GSEA and GSVA

To further elucidate the mechanistic differences between the TED and normal groups, we included all the DEGs in gene set enrichment analysis (GSEA) [40]. GESA offered three benefits over conventional enrichment: 1) genes were ranked according to their expression levels; 2) the object of analysis was a gene set rather than a single gene; and 3) the genes were compared to a predefined gene set. According to the annotation and description, we selected and downloaded reference data for two gene sets, “c2.cp.kegg.v2022.1.Hs.symbols.gmt” and “c5.go.all.v2022.1.Hs.symbols.gmt”, from the Human Molecular Signatures Database (MSigDB) [41]. The results are displayed as biological process (BP), cell component (CC), molecular function (MF), and KEGG. For visualization, classic and ridge diagrams were employed, with the clusterProfiler package [42] in R used to calculate the normalized enrichment score (NES) and false discovery rate (FDR).

Gene set variation analysis (GSVA), a nonparametric unsupervised algorithm, transforms a gene-sample expression matrix into a specific gene set-sample expression matrix [43]. Using this strategy, we sorted the genes based on their cumulative density distribution and computed the rank statistics for further downstream analysis. The MSigDB gene sets “c2.cp.kegg.v2022.1.Hs.symbols.gmt” and “c5.go.all.v2022.1.Hs.symbols.gmt” were incorporated in GSVA, and the route of enrichment differences between the test and control groups were compared as a result.

### WGCNA

Weighted gene correlation network analysis (WGCNA) is defined as a systematic biological technique for characterizing gene relationship patterns across samples. By examining the interrelation between the gene set and its associations with other phenotypes, it may be used to identify gene sets with significant covariation as well as possible biomarker genes or therapeutic targets [44]. We used this method to build a gene co-expression network and discovered essential modules consisting of the interconnected gene sets in both TED and normal samples.

### Protein-protein interaction (PPI) network and hub gene identification

We extracted the most relevant gene modules from WGCNA and used them to interact with DEGs. The DEG collection was then imported into the “multiple proteins” module of the STRING database (http://string-db.org/) [45], which allows functional proteomic interaction analysis. The main parameters were set as follows: active interaction sources (“Text mining and Experiments and Databases”), max number of interactors to show [“1st shell: no more than 50 interactors”], minimum required interaction score [“medium confidence (0.400)”] and others were left at default. Next, we built the TED-related PPI network using Cytoscape (version 3.9.1) [46, 47], a free program for network data integration and visualization. We investigated the TED-related hub genes further in Cytoscape using the MCC algorithm of the CytoHubba module [48]. The top ten hub gene networks were categorized, as were the top five hub gene networks discovered by the MCODE module [49].

We used ridge diagrams to show the distribution of the aforesaid hub genes (H-DEGs) in TED and normal tissues according to their expression levels. Moreover, the diagnostic capability of these genes was evaluated by employing ROC and AUC.

### Correlation analysis of immune cell infiltration

While the importance of the immune response in TED has been acknowledged, limited research has delved into the involvement of immune cells and inflammatory factors in the disease and their specific pathways. It is crucial to conduct immune infiltration studies at TED to uncover novel mechanisms and potential therapeutic targets related to immunity. To estimate the abundance of different cell types within a mixed population based on gene expression profiles, we utilized CIBERSORTx, an analytical tool (https://cibersortx.stanford.edu/) [50]. This program was employed to calculate the levels of 22 immune cell types in both the TED and normal groups. The distribution and infiltration abundance of these 22 immune cells between the test and control groups were visually represented using box plots and histograms. Additionally, we examined how F-DEGs and H-DEGs relate to immunocytes as well as their interaction with both TED and normal tissues.

### DEG identification and correlation analysis based on thyroid samples

Aside from lacrimal gland samples, we further included thyroid samples in this study by downloading the raw data from dataset GSE9340 [17]. After the data normalization procedure, 10 TED and 8 normal samples were included in the subsequent analysis. GEO2R was utilized to screen the top DEGs with a threshold of |log2-fold change (FC)| ≥ 1 and a *p*-value < 0.05. We utilized Pearson’s method to determine the correlation coefficients and depict the association based on the data matrix to further investigate the correlation of the screened DEGs between thyroid and lacrimal gland tissues.

### Experimental validation of DEG expression and immune infiltration

Based on the orbital CT scans obtained from both the TED and normal groups, a thorough comparison was made to evaluate the degree of adipose infiltration and ocular muscle hypertrophy in each group. To further investigate these findings, 23 pairs of periorbital adipose samples were selected for real-time quantitative PCR (RT-qPCR) and western blotting (WB) analyses (details of characteristics were summarized in Supplementary Tables 1, 2). These tissue samples were taken via endoscopic resection and immediately stored in liquid nitrogen to maintain their integrity and biological activity.

RT-qPCR: A prior protocol [51] was followed for tissue isolation and total RNA extraction. Briefly, quality-controlled RNA was converted to cDNA using the PrimeScript™ RT reagent kit with gDNA Eraser (cat#RR047A; Takara, Japan) and amplified using the SYBR® Premix Ex Taq™ II kit (cat#RR820A; Takara) on the ABI PRISM® 7500 Sequence Detection System (Applied Biosystems, CA, USA) following the manufacturer’s protocols. Table 1 displays the primer sequences generated by Primer 6.0 (Applied Biosystems, CA, USA). The mixture was incubated for 30 seconds at 95° C, followed by 40 cycles of 5 seconds at 95° C and 34 seconds at 60° C. The 2^−ΔΔCT^ approach was used to perform semiquantitative gene expression analysis with normalized levels of the housekeeping gene glyceraldehyde 3-phosphate dehydrogenase (GAPDH).

**Table 1:** Primers designed for RT-qPCR.

**Gene**	**F:5’-3’**	**R:5’-3’**
GAPDH	ACCCACTCCTCCACCTTTGAC	TCCACCACCCTGTTGCTGTAG
MYH11	AGCCAGAGACGAGAGGACATTC	GGAGAGGAAGGTGTAGTTGTTGAAG
APOD	GCCACCGACTATGAGAACTATGC	ACTGTTTCTGGAGGGAGATTAGGG
EGR2	CTCCTCCTCCTTATTCTGGCTGTG	GGTCCGTGGCTGGCTTGG
IDH1	CACCAACGACCAAGTCACCAAG	ACTCCTCAACCCTCTTCTCATCAG
PEX3	TTGCGGGTCCAGTTAAACATAATTG	CCATCTCCAAGTAGGTGCTGAATAC
MYCN	AGAAGCGGCGTTCCTCCTC	GTTGTGCTGCTGGTGGATGG
EMC2	AGACTATGGTCGGGATGACTTGG	ATGCCTGTTAATCGCTTGACTCTG
CDH1	GCTAATTCTGATTCTGCTGCTCTTG	GTCCTCTTCTCCGCCTCCTTC
MDM4	AGACCCAAGCCCTCTCTATGATATG	AGAGTCTGAGCAGCATCTGTAGTAG
CP	GGTCCAGGAGTGTAACAAGTCTTC	CCTCAGCGGCAATGTAGTAGTG
TF	ATCAGCAGAGACCACCGAAGAC	ACAGGCACCAGACCACACTTG
OSBPL9	AGTTGGACCTGTGTTGGCTACC	ACTGCTACTCGGTGGTGAATGG
DNAJB6	TGCCTCGCTGCTGAGACAC	CTCTGCTTCTGCTTCTTCCTCTTG
TFAP2A	AATGCCGTCTCCGCCATCC	TTCCGCCACCGTGACCTTG
YKT6	CCGCATACGATGTGTCTTCCTTC	CGCTCCACAATCAGTTGACTCG
ITCH	ACCTTCACGACCACCACCAC	AATCCAGATGTTGCTCCTTCAGATG
USO1	CACACAGAAGCCGAGACGATTC	GATTTGAGAGCACGAACAGCATTTC
SRSF1	TACCTCCAGACATCCGAACCAAG	CGAACTCAACGAAGGCGAAGG
PSMA3	GCTCAATCGGCACTGGGTATG	CCACAGCCTTCATAGCATATTCAAC
SNRPG	TGATAGATGAATGTGTGGAGATGGC	ACTTTGGGCTTACCGCTTTATTAGG
COPB2	GTTGTGACAGGAGCGGATGAC	GGATGAACAGCAATACAGCGAATG
PSMD6	AGGAGCAGAGATTCTTGAAGTGTTG	ACAGAGTAACGGCATTCATAGAGTG
PSMA4	CAGAGAGACGCAACATCCACAAG	ACCTTTGAGCAATGAGCCTTAGTTC
RBM25	GGAAGAGGAAGAGGAGCCAGAG	CAGAGGAAACAGATGGAGCAGAG
PSMD14	GCTATGCCACAGTCAGGAACAG	TGATACCAACCAACAACCATCTCC
LMAN1	GACAACACAGCACTTCATTGACATC	GGTGGTAGTTCTGGGCATTTCG
PSMD12	AGGTGGAAAGACTTGAAGAACAGAG	GGCTTCGGACTCATCAACAGATAG
PSMA1	GGCTTACTGCTGATGCTAGACTG	TTAGAGATACAAGACGAGACACAGG
RAB1A	TGTCCAGCATGAATCCCGAATATG	AGAAGAAGGCAAGACTTTCCAACC

WB: Using radioimmunoprecipitation assay (RIPA) buffer (Beyotime Biotechnology, Shanghai, China) and a BCA protein assay kit (Beyotime Biotechnology, Shanghai, China), the total proteins were extracted and quantitated. Targeted proteins were separated using 10% sodium dodecyl sulfate-polyacrylamide gel electrophoresis (SDS-PAGE) and subsequently transferred to polyvinylidene difluoride (PVDF) membranes (Millipore, MA, USA). The membranes were blocked for 2 hours at ambient temperature using a 5% fat-free milk solution in Tris-buffered saline containing 0.05% Tween-20. Subsequently, the membranes were subjected to an overnight incubation at 4° C with primary antibodies (details of antibodies were summarized in Supplementary Table 3). Corresponding secondary antibodies were then coincubated at room temperature for 1 hour after being appropriately diluted in PBS. The protein bands were visualized using chemiluminescence and quantified using ImageJ software, following coating with the ECL assay kit (EpiZyme, China).

Immunohistochemistry (IHC): Immunohistochemical analysis was employed to investigate immune cell infiltration in six pairs of TED patients’ periorbital lipid tissues. The standard procedures were performed as published [52]. Frozen sections were fixed in methanol for 20 minutes in total. Following antigen retrieval and suppression of endogenous peroxidase activity, the sections were sealed for 30 minutes at room temperature with 3% BSA (Sigma, CA, USA). After the blocking solution was removed, preconfigured primary antibodies were added to the sections for overnight coincubation at 4° C. Subsequently, the sections were incubated at room temperature for 50 minutes with the corresponding secondary antibodies (details of antibodies were summarized in Supplementary Table 3). For section staining, DAB chromogenic solution (Genetech, Shanghai, China) and hematoxylin were used (the nucleus of hematoxylin stained is blue, and the positive expression of DAB is brownish yellow). An imaging system (Leica DFC450C, Leica, Shanghai, China) was used for image acquisition and analysis.

### Statistical analysis

R software (version 4.2.1) was used for all statistical analyses and graphing. The t-test and one-way ANOVA were employed to assess variables with a normal distribution, while nonparametric tests were utilized for variables that did not follow a normal distribution. Survival analysis was conducted using the log-rank test and Cox regression, whereas Pearson’s correlation and Spearman’s rank correlation test were employed for correlation analysis. Statistical significance was defined as a *p*-value of 0.05. Correlation ranges were established as follows: 0.00-0.10 (negligible), 0.10-0.39 (weak), 0.40-0.69 (moderate), 0.70-0.89 (strong), and 0.90-1.00 (extremely strong) [53].

### Availability of data and materials

The original contributions presented in the study are included in the article/Supplementary Material, and further inquiries can be directed to the corresponding author(s).

All data and original files in our work are freely available under a ‘Creative Commons BY 4.0’ license. All methods were carried out in accordance with relevant guidelines and regulations.

## RESULTS

**Figure 1:**
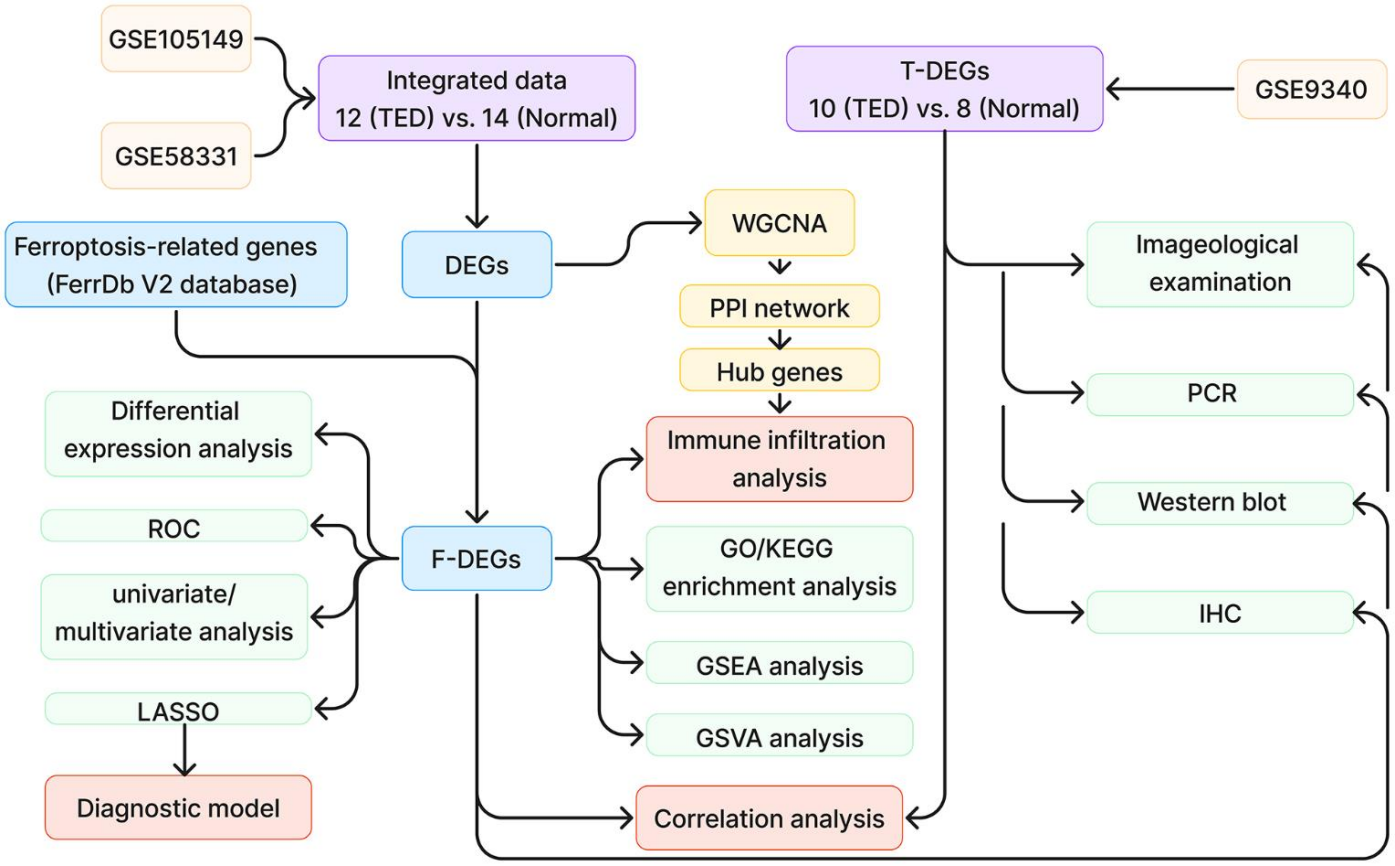
**Study design flow chart.** TED, thyroid eye disease; DEGs, differentially expressed genes; ROC, receiver operating characteristic; WGCNA, weighted correlation network analysis; PPI, protein-protein interaction network; GO, Gene Ontology; KEGG, Kyoto Encyclopedia of Genes and Genomes; GSEA, gene set enrichment analysis; GSVA, gene set variation analysis; IHC, immunohistochemistry.

Figure 1 demonstrates the whole design and procedure of this study.

### Data normalization

**Figure 2:**
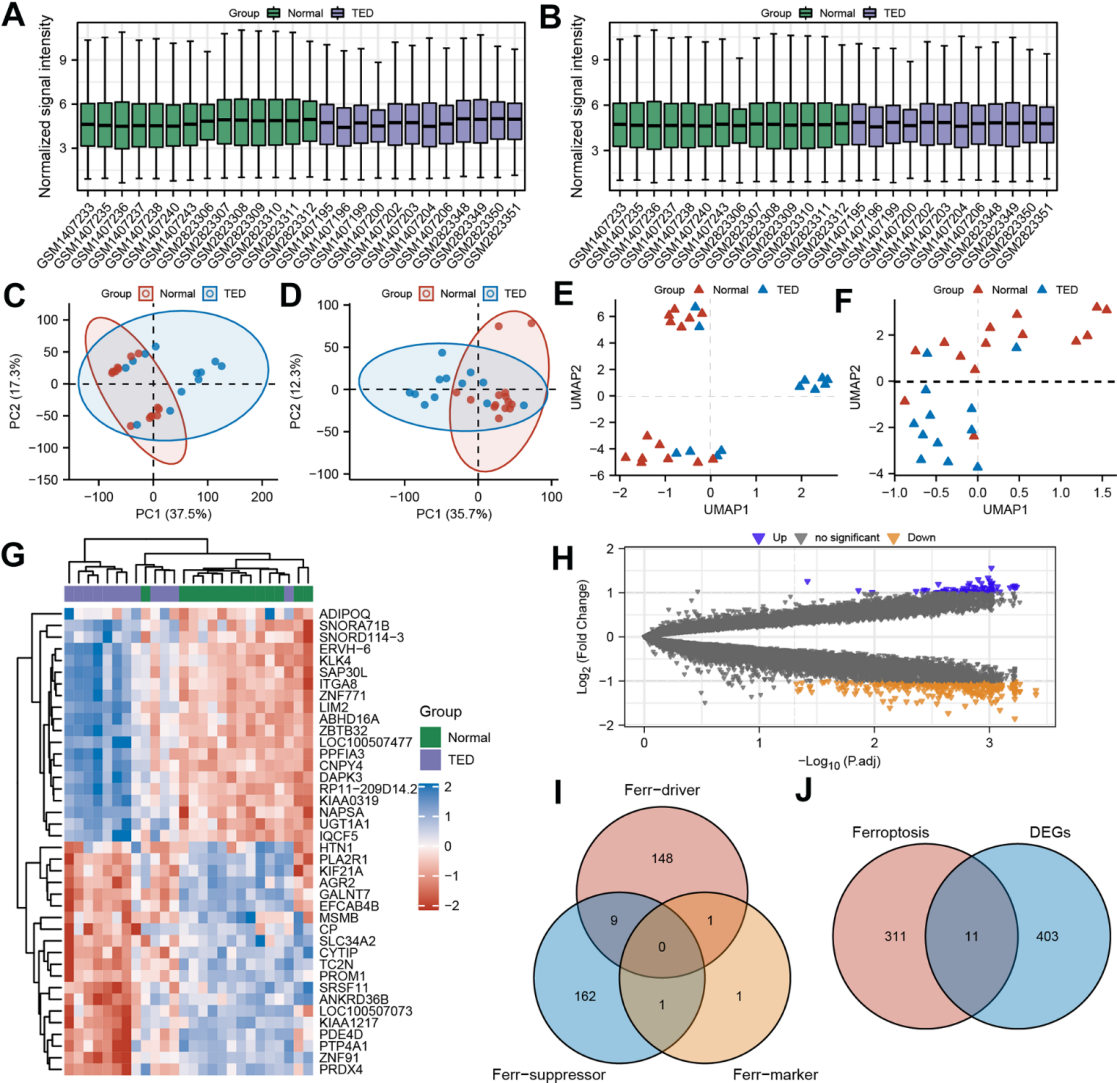
**Screening of ferroptosis-related DEGs (F-DEGs) in the lacrimal gland.** (**A**, **B**) Box plot of gene expression before and after batch effect removal in the integrated GEO data; (**C**, **D**) PCA of gene expression before and after batch effect removal in the integrated GEO data; (**E**, **F**) UMAP of gene expression before and after batch effect removal in the integrated GEO data; (**G**) Heatmap of DEGs; (**H**) Volcano plots of DEGs; (**I**) Venn diagram of ferroptosis-related genes; (**J**) Venn diagram of the intersection of ferroptosis-related genes and DEGs. DEGs, differentially expressed genes; GEO, Gene Expression Omnibus; PCA, principal component analysis; UMPA, uniform manifold approximation and projection.

To comprehensively evaluate and analyse variations in gene expression, we combined and merged two distinct datasets. We implemented a standardized process to remove batch effects and normalize the original data, enhancing the accuracy and efficiency of subsequent data analysis. The processed data exhibited significantly superior standardized signal intensity compared to the initial data processing stage (Figure 2A, 2B). Using principal component analysis (PCA), the standardized data exhibited improved within-group repeatability and between-group discriminability (Figure 2C, 2D). The uniform manifold approximation and projection (UMAP), a technique for reducing dimensions, showed consistent results regarding the data distribution as above (Figure 2E, 2F).

### F-DEG screening

A total of 12 TED samples and 14 control samples comprised the consolidated data. After examining the variations in gene expression among the two groups, 414 DEGs were screened by applying the filter criterion |Log_2_FC| > 1 and *p* < 0.05. The results are depicted using a heatmap (Figure 2G), which displays the panorama and clusters of DEGs, with 73 upregulated genes and 341 downregulated genes further visualized as a volcano diagram (Figure 2H).

**Figure 3:**
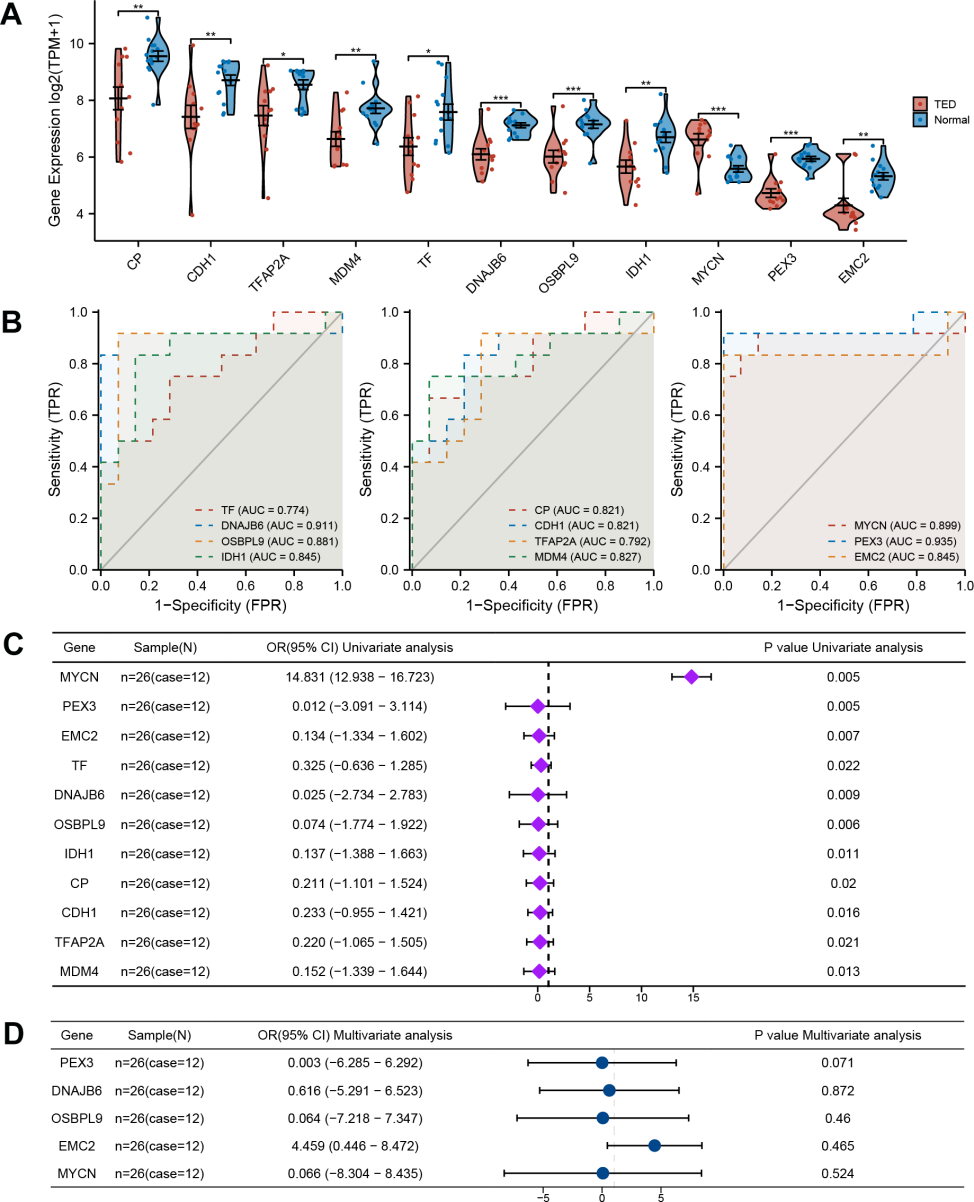
**Comprehensive evaluation of F-DEGs.** (**A**) Gene expression analysis of F-DEGs between TED and normal groups; (**B**) Diagnostic value analysis of F-DEGs; (**C**) Univariate analysis of F-DEGs; (**D**) Multivariate analysis of F-DEGs. *P < 0.05, **P < 0.01, ***P < 0.001.

Through the integration of ferroptosis driver, ferroptosis suppressor, and ferroptosis marker gene sets, 322 ferroptosis-related genes were obtained (Figure 2I). By further intersecting with the DEGs above, we identified 11 ferroptosis-related DEGs (F-DEGs) (Figure 2J). The expression patterns of these 11 F-DEGs were detailed and are shown in a violin plot (CP, CDH1, TFAP2A, MDM4, TF, DNAJB6, OSBPL9, IDH1, MYCN, PEX3, and EMC2) (Figure 3A). MYCN stood out as a gene that was specifically overexpressed in the TED group (*p* < 0.001), while the majority of genes showed increased expression in the normal group (all *p* < 0.05) (Figure 3A).

### Clinical value assessment of F-DEGs

Additionally, we conducted an evaluation to ascertain the diagnostic value of the F-DEGs for TED. The findings showed that every single F-DEG exhibited exceptional diagnostic performance for TED (TF: AUC = 0.774, DNAJB6: AUC = 0.911, OSBPL9: AUC = 0.881, IDH1: AUC = 0.845, CP: AUC = 0.821, CDH1: AUC = 0.821, TFAP2A: AUC = 0.792, MDM4: AUC = 0.827, MYCN: AUC = 0.899, PEX3: AUC = 0.935, and EMC2: AUC = 0.845) (Figure 3B).

We further performed a univariate logistic analysis of F-DEGs to examine their associations with TED diagnosis. In the diagnosis of individuals with TED, MYCN was identified as a significantly important independent factor (OR = 14.831, 95% confidence interval (CI) = 12.938-16.723, *p* = 0.005). The examination of other F-DEGs, on the other hand, revealed a possibly unfavorable effect on the diagnosis of TED (all OR < 1, *p* < 0.05) (Figure 3C). Genes that had a statistical significance of *p* < 0.01 were chosen for inclusion in the multivariate Cox regression analysis. EMC2 potentially had a favorable association with TED (OR = 4.459, 95% CI = 0.446-8.472). PEX3 (OR =0.003, 95% CI = -6.285-6.292), DNAJB6 (OR = 0.616, 95% CI = -5.291-6.523), OSBPL9 (OR = 0.064, 95% CI = -7.218-7.347), and MYCN (OR = 0.066, 95% CI = -8.304-8.435), on the other hand, revealed an inverse correlation (Figure 3D). However, no statistical significance supported these correlations (all *p* > 0.05).

### Establishment and validation of the diagnostic prediction model

**Figure 4:**
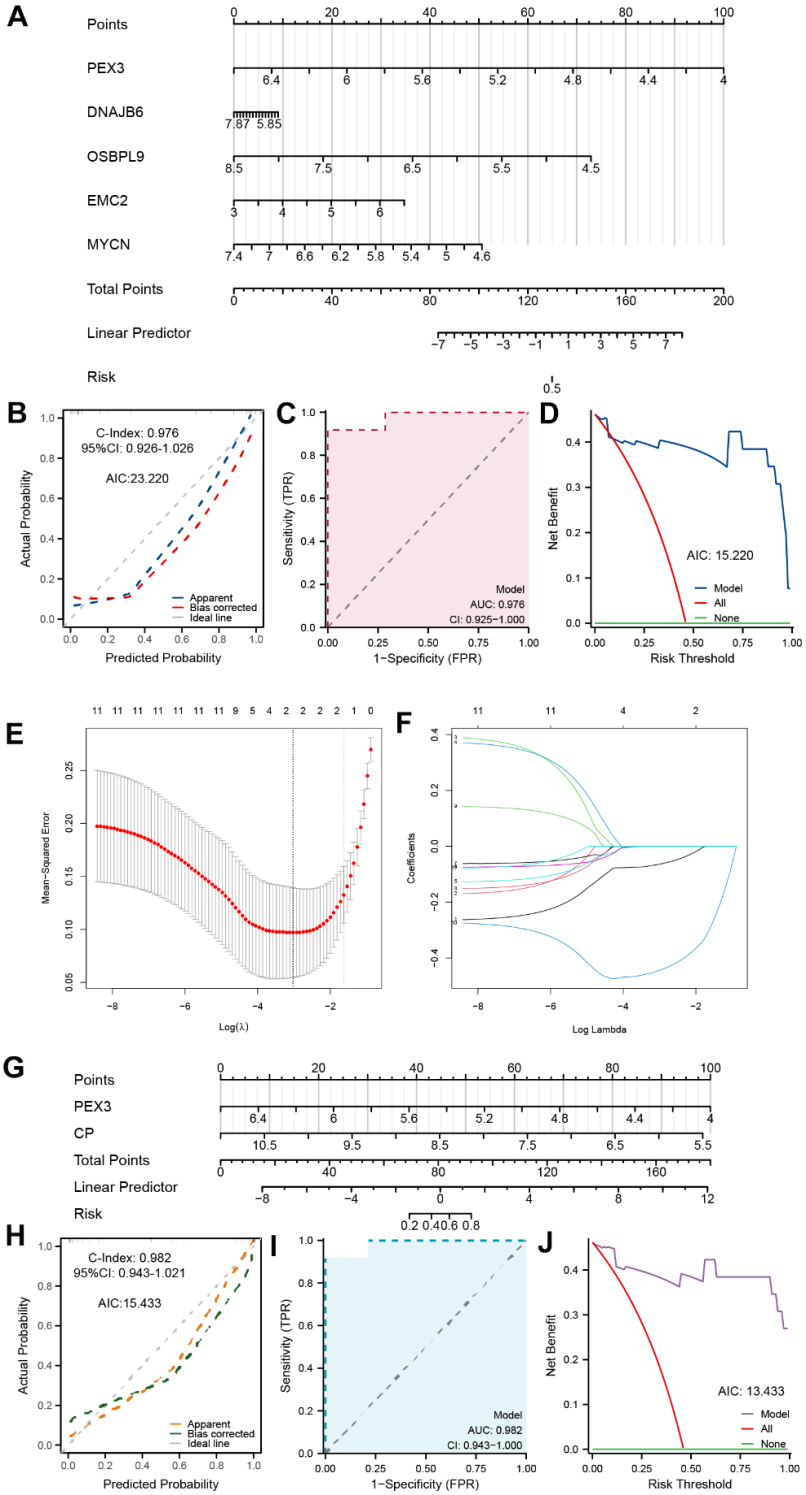
**Establishment of a ferroptosis-related TED model.** (**A**) Diagnostic nomogram of ferroptosis-related TED; (**B**) Nomogram calibration curve of the diagnostic model; (**C**) ROC curve of the diagnostic model; (**D**) Decision curve for evaluating the net benefits of the nomogram; (**E**) Further identification of F-DEGs via LASSO regression; (**F**) Trajectory chart of LASSO regression; (**G**) Diagnostic nomogram of ferroptosis-related TED by LASSO; (**H**–**J**) Nomogram calibration curve, ROC curve, and decision curve for evaluating the precision and robustness of the diagnostic model. *P < 0.05, **P < 0.01, ***P < 0.001.

Based on the above results, we first integrated the five most statistically relevant genes and constructed a ferroptosis-related nomogram for TED risk prediction (Figure 4A). PEX3 made the greatest contribution and was recognized as an important independent diagnostic factor in addition to the other four factors. This model demonstrated good accuracy and robustness in providing diagnostic prediction scores for TED patients’ clinical episodes (C-index = 0.976, 95% CI = 0.926-1.026, AIC = 23.220) (Figure 4B). We supplementally employed a time-dependent ROC curve to evaluate the diagnostic precision of this model (AUC = 0.976, 95% CI = 0.925-1.000) (Figure 4C) and performed DCA to assess the practicality of this model (AIC = 15.220) (Figure 4D).

Furthermore, we adopted a machine learning strategy (LASSO regression) to filter the 11 F-DEGs for the most diagnostically valuable genes. The LASSO coefficients were calculated using a tenfold cross-verification procedure and presented as a variable trajectory diagram (Figure 4E, 4F). We subsequently established a new F-DEG-related diagnostic nomogram that included PEX3 and CP, both of which contributed equally to this prediction model (Figure 4G). This model’s excellent predictive ability was confirmed (C-index = 0.982, 95% CI = 0.943-1.021, AIC = 15.433) (Figure 4H). The model’s precision and dependability were further confirmed by the time-dependent ROC curve (AUC = 0.982, 95% CI = 0.943-1.000) (Figure 4I) and DCA (AIC = 13.433) (Figure 4J).

### Biological enrichment analysis

**Figure 5:**
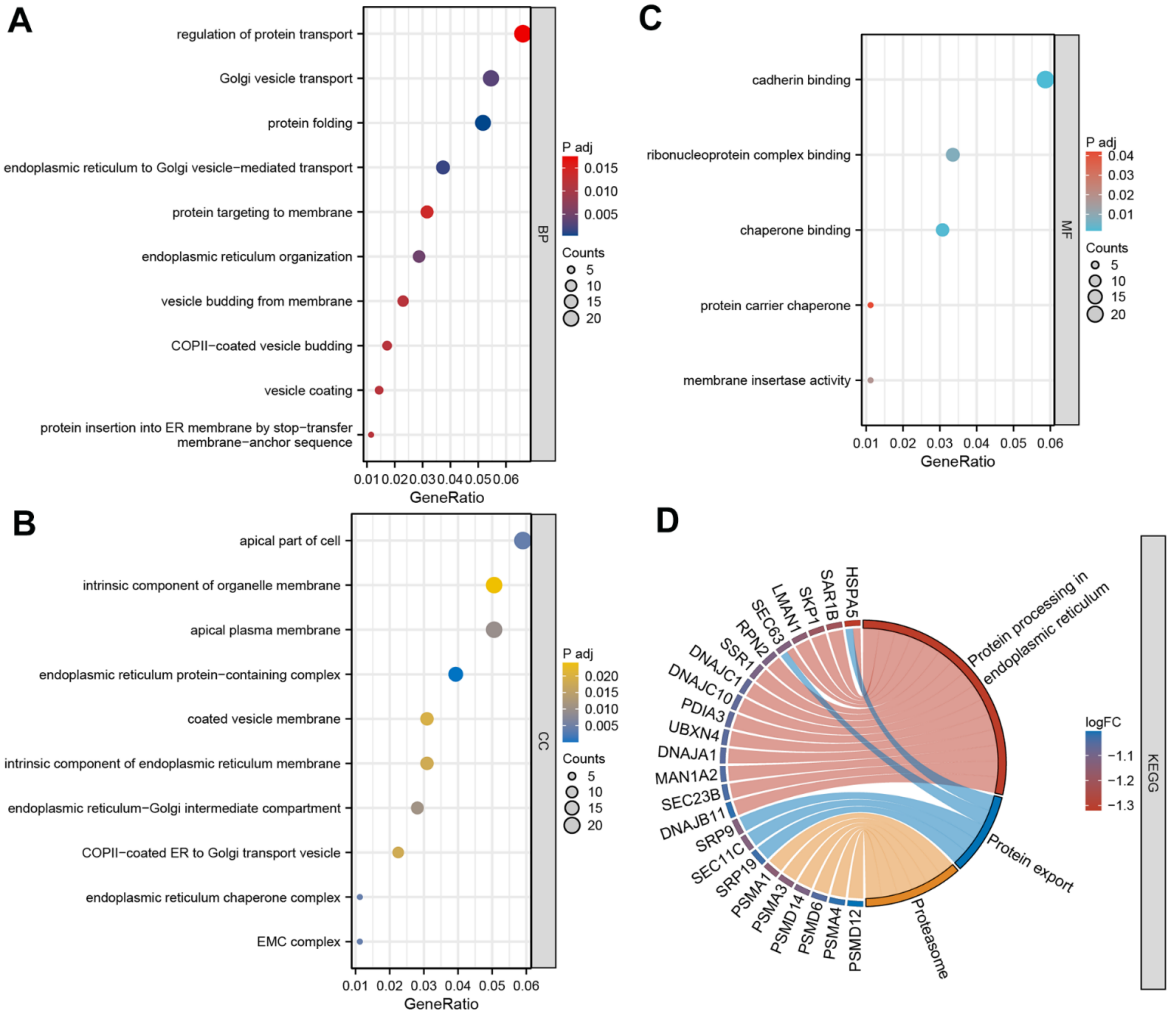
**GO and KEGG enrichment analysis.** (**A**) Bubble diagram of BP enrichment analysis; (**B**) Bubble diagram of CC enrichment analysis; (**C**) Bubble diagram of MF enrichment analysis; (**D**) Chord diagram of KEGG pathway enrichment analysis. BP, biological process; CC, cell component; MF, molecular function. *P < 0.05, **P < 0.01, ***P < 0.001.

To delve deeper into the molecular mechanism of DEGs in TED, we employed GO and KEGG enrichment analyses. By setting a significance threshold of *p* < 0.05, we successfully identified the top 10 correlated pathways of BP and CC, the top 5 correlated pathways of MF, and the top 3 correlated pathways of KEGG. “Regulation of protein transport”, “Golgi vesicle transport”, “protein folding”, “endoplasmic reticulum to Golgi vesicle-mediated transport”, “protein targeting to membrane”, “endoplasmic reticulum organization”, “vesicle budding from membrane”, “COPII-coated vesicle budding”, “vesicle coating”, and “protein insertion into ER membrane by stop-transfer membrane-anchor sequence” of BP (all *p* < 0.001) were mainly involved in TED (Figure 5A). Consistently, CC pathways were also primarily associated with protein coating and membrane transport (all *p* < 0.001) (Figure 5B). Regarding MF, we noticed an enrichment of DEGs in the following pathways: “cadherin binding”, “ribonucleoprotein complex binding”, “chaperone binding”, “protein carrier chaperone”, and “membrane insertase activity” (all *p* < 0.001) (Figure 5C). The KEGG pathways showed the strongest association with “protein processing in endoplasmic reticulum”, “protein export”, and “proteasome” (all *p* < 0.001) (Figure 5C).

**Figure 6:**
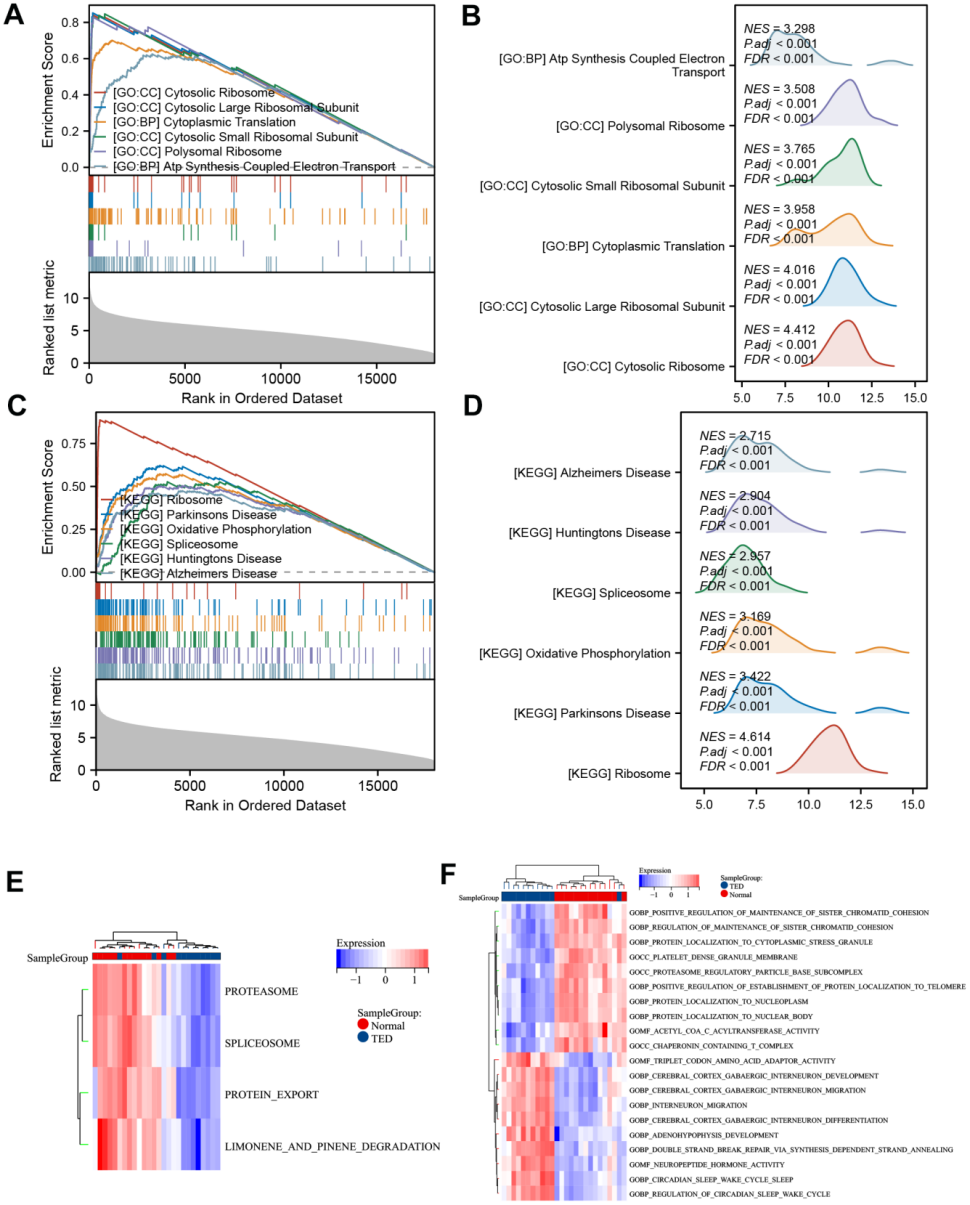
**GSEA and GSVA.** (**A**) GSEA-GO of DEGs; (**B**) ridge plot of top 6 GSEA-GO; (**C**) GSEA-KEGG of DEGs; (**D**) ridge plot of top 6 GSEA-KEGG; (**E**) heatmap of functional scores of GSVA-KEGG; (**F**) heatmap of functional scores of GSVA-GO. *P < 0.05, **P < 0.01, ***P < 0.001.

We employed another algorithm to explore the between-group differences in DEG-enriched pathways. The enriched bioprocesses were ranked according to their enrichment score (Figure 6A). The top 6 GO pathways were involved in “ATP synthesis coupled electron transport” (NES = 3.298, *p* adj < 0.001), “polysomal ribosome” (NES = 3.508, *p* adj < 0.001), “cytosolic small ribosomal subunit” (NES = 3.765, *p* adj < 0.001), “cytoplasmic translation” (NES = 3.958, *p* adj < 0.001), “cytosolic large ribosomal subunit” (NES = 4.016, *p* adj < 0.001), and “cytosolic ribosome” (NES = 4.412, *p* adj < 0.001) (Figure 6B). On the other hand, the top 6 KEGG pathways included “Alzheimer’s disease” (NES = 2.715, *p*.adj < 0.001), “Huntington’s disease” (NES = 2.904, *p*.adj < 0.001), “spliceosome” (NES = 2.957, *p*.adj < 0.001), “oxidative phosphorylation” (NES = 3.169, *p*.adj < 0.001), “Parkinson’s disease” (NES = 3.422, *p*.adj < 0.001), and “ribosome” (NES = 4.614, *p*.adj < 0.001) (Figure 6C, 6D).

The possible trend of association between enriched pathways and TED was discovered further with the use of GSVA. In TED samples, the KEGG pathways “proteasome”, “spliceosome”, “protein export”, and “limonene and pinene degradation” were all restricted (Figure 6E). The findings additionally indicated that TED exhibited primarily positive associations with neural bioprocesses such as “cerebral cortex GABAergic interneuron”, “neuropeptide hormone activity”, and similar processes. Conversely, TED displayed negative correlations with protein and chromatid bioprocesses such as “protein localization to nucleoplasm” and “regulation of sister chromatid cohesion” (Figure 6F).

### WGCNA and hub gene network

**Figure 7:**
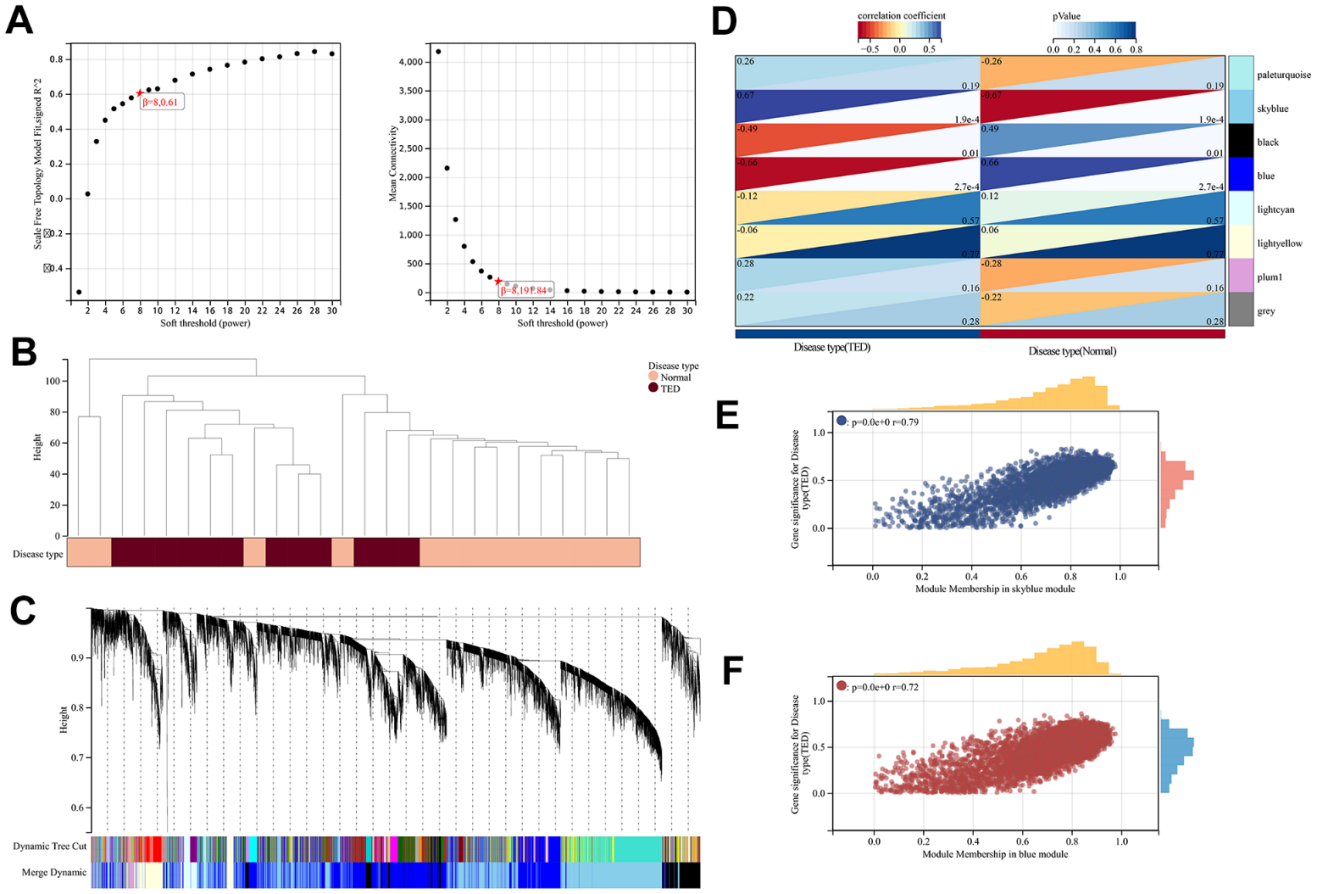
**WGCNA.** (**A**) WGCNA of soft threshold screening; (**B**) Sample clustering and disease type; (**C**) Co-expression gene clustering; (**D**) Correlation analysis between co-expression gene modules and clinical characteristics; (**E**) Correlation analysis between MM and GS in the sky blue module; (**F**) Correlation analysis between MM and GS in the blue module. MM, module membership; GS, gene significance. *P < 0.05, **P < 0.01, ***P < 0.001.

To identify gene sets with high synergistic variation, we subjected all genes to WGCNA. We excluded the bottom 50% of the genes with the lowest median absolute deviation (MAD), eliminated any genes or samples that were outliers, and built a co-expression network that follows a scale-free pattern (Figure 7A). Sample and gene clusters are displayed in Figure 7B, 7C, respectively. Then, to consolidate similarity and decrease redundancy, we assessed the dissimilarity of module eigengenes and merged some modules. Following these steps, we successfully obtained a total of eight co-expression modules. Based on the correlation analysis, the blue module containing 4591 genes was most negatively correlated with TED (cor = -0.66, *p* = 2.7e-4), whereas the skyblue module, which had 3775 genes, was most positively correlated with TED (cor = 0.67, *p* = 1.9e-4) (Figure 7D). Furthermore, we evaluated the correlation between gene significance (GS) and module membership (MM). To be consistent, a high degree of synergy of genes within each module was observed, especially in the skyblue (cor = 0.76, *p* < 0.001) (Figure 7E) and blue modules (cor = 0.72, *p* < 0.001) (Figure 7E).

**Figure 8:**
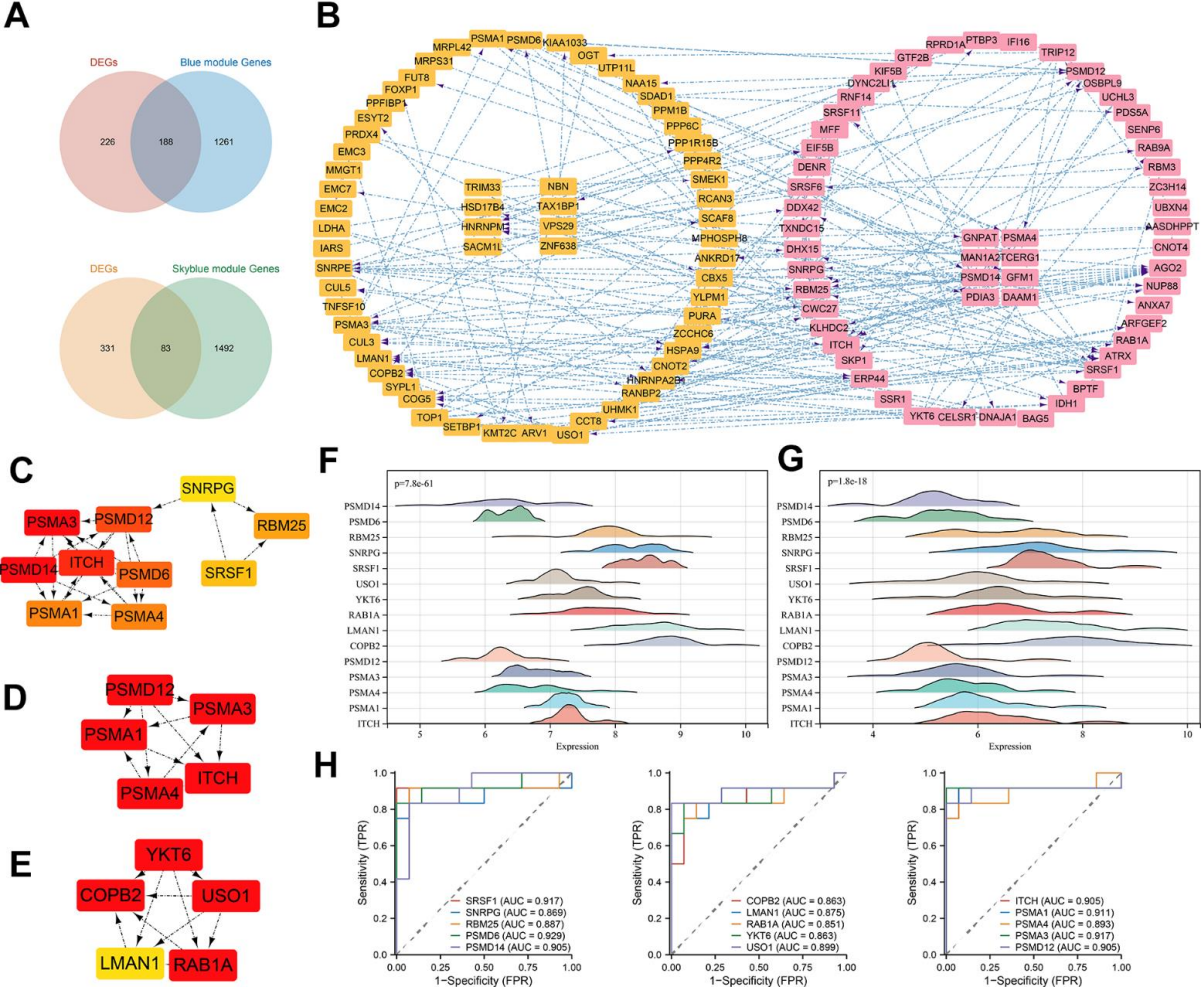
**PPI construction and hub gene identification.** (**A**) Venn diagram of intersection of DEGs and most significant module genes; (**B**) PPI network of key genes in blue module; (**C**) Top 10 hub genes by MCC algorithm; (**D**) Top 5 hub genes of cluster 1; (**E**) Top 5 hub genes of cluster 2; (**F**) 15 hub genes expression distribution in normal samples; (**G**) 15 hub genes expression distribution in TED samples; (**H**) ROC curve of 15 hub genes. *P < 0.05, **P < 0.01, ***P < 0.001.

Next, we intersected the above two gene set modules with the DEGs and obtained 83 DEGs for the skyblue module and 188 DEGs for the blue module (Figure 8A). Based on the evidence from text mining, experiments, and databases, we constructed DEG-related PPI networks of 50 predicted functional partners (Figure 8B). Additionally, we selected the whole interaction network and the top 2 clusters from the DEGs to mine for potential hub genes. Using the MCC algorithm, the top 10 hub genes, including PSMA1, PSMA4, PSMA3, PSMA12, PSMA14, PSMA6, ITCH, SNRPG, SRSF1, and RBM25, were screened from the whole interaction network (Figure 8C). The top 5 hub genes of Cluster 1 (score = 7.000) were PSMA1, PSMA4, PSMA3, PSMA12, and ITCH (Figure 8D). Meanwhile, the top 5 hub genes of YKT6, COPB2, USO1, LMAN1, and RAB1A were included in Cluster 2 (score = 5.600) (Figure 8E).

A total of 15 hub gene-related DEGs (H-DEGs) were integrated. Ridge diagrams fully displayed the distribution differences of H-DEG expression in the TED (*p* = 7.8e-61) (Figure 8F) and normal groups (*p* = 1.8e-18) (Figure 8G). We further performed ROC curves to evaluate the diagnostic capacity of H-DEGs in TED. A remarkable accuracy of all H-DEGs in diagnosis was observed: SRSF1 (AUC = 0.917), SNRPG (AUC = 0.869), RBM25 (AUC = 0.887), PSMD6 (AUC = 0.929), PSMD14 (AUC = 0.905), COPB2 (AUC = 0.863), LMAN1 (AUC = 0.875), RAB1A (AUC = 0.851), YKT6 (AUC = 0.863), USO1 (AUC = 0.899), ITCH (AUC = 0.905), PSMA1 (AUC = 0.911), PSMA4 (AUC = 0.893), PSMA3 (AUC = 0.917), and PSMA12 (AUC = 0.905) (Figure 8H).

### Immune infiltration analysis

**Figure 9:**
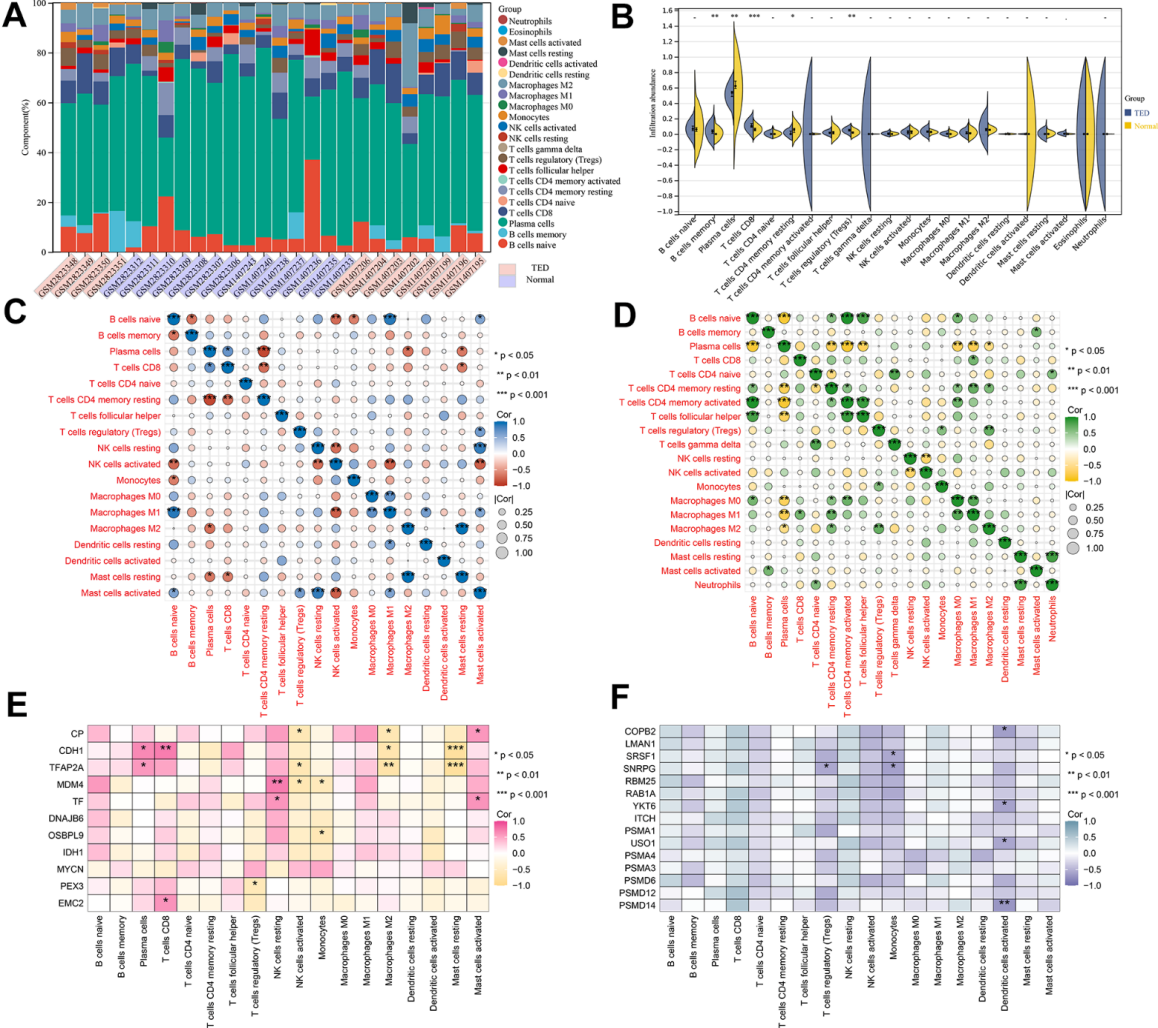
**Immune infiltration analysis.** (**A**) Accumulative immune cell concentrations in the TED and normal groups; (**B**) Analysis of different immune cell infiltration between TED and normal groups; (**C**) Correlation analysis of immune cell infiltration in the TED group; (**D**) Correlation analysis of immune cell infiltration in the normal group; (**E**) Correlation analysis between F-DEGs and immune cells; (**F**) Correlation analysis between 15 hub genes and immune cells. *P < 0.05, **P < 0.01, ***P < 0.001.

We obtained 22 immune cell infiltration scores for lacrimal samples via the CIBERSORT algorithm. Regardless of whether TED or normal samples were taken, the component of plasma cells was the highest (Figure 9A). According to the infiltration abundance, the TED group had a higher degree of B-cell memory (*p* < 0.01), CD8 T cells (*p* < 0.001), and regulatory T cells (Tregs) (*p* < 0.01) infiltration than the normal group. In contrast, the infiltration levels of plasma cells (*p* < 0.01) and resting memory CD4 T cells (*p* < 0.05) were higher in the normal group than in the TED group (Figure 9B). Furthermore, we examined the interactions of immune cells in lacrimal tissue, as well as their interactions with F-DEGs and H-DEGs. In the TED group, there was a strong positive correlation between M1 macrophages and naive B cells, resting mast cells and M2 macrophages, and activated mast cells and resting NK cells, while there was a strong negative correlation between resting memory CD4 T cells and plasma cells (all *p* < 0.001) (Figure 9C). In the normal group, we found a strong positive correlation between activated memory CD4 T cells/nerve follicular helper T cells and naive B cells, neutrophils and resting mast cells and a strong negative correlation between plasma cells and naive B cells/activated memory CD4 T cells (all *p* < 0.001) (Figure 9D). Furthermore, the findings demonstrated a close relationship between F-DEGs and immune cell infiltration. In particular, CDH1 and TFAP2A were strongly negatively correlated with resting mast cells (all *p* < 0.001). There was also a strong positive link between resting NK cells and MDM4, as well as CD8 T cells and CDH1 (all *p* < 0.01) (Figure 9E). In the case of H-DEGs, no significantly positive correlation with immune cells was identified; however, a significantly negative connection between PSMD14 and activated dendritic cells was observed (*p* < 0.01) (Figure 9F).

### Correlation analysis of DEGs in thyroid samples

**Figure 10:**
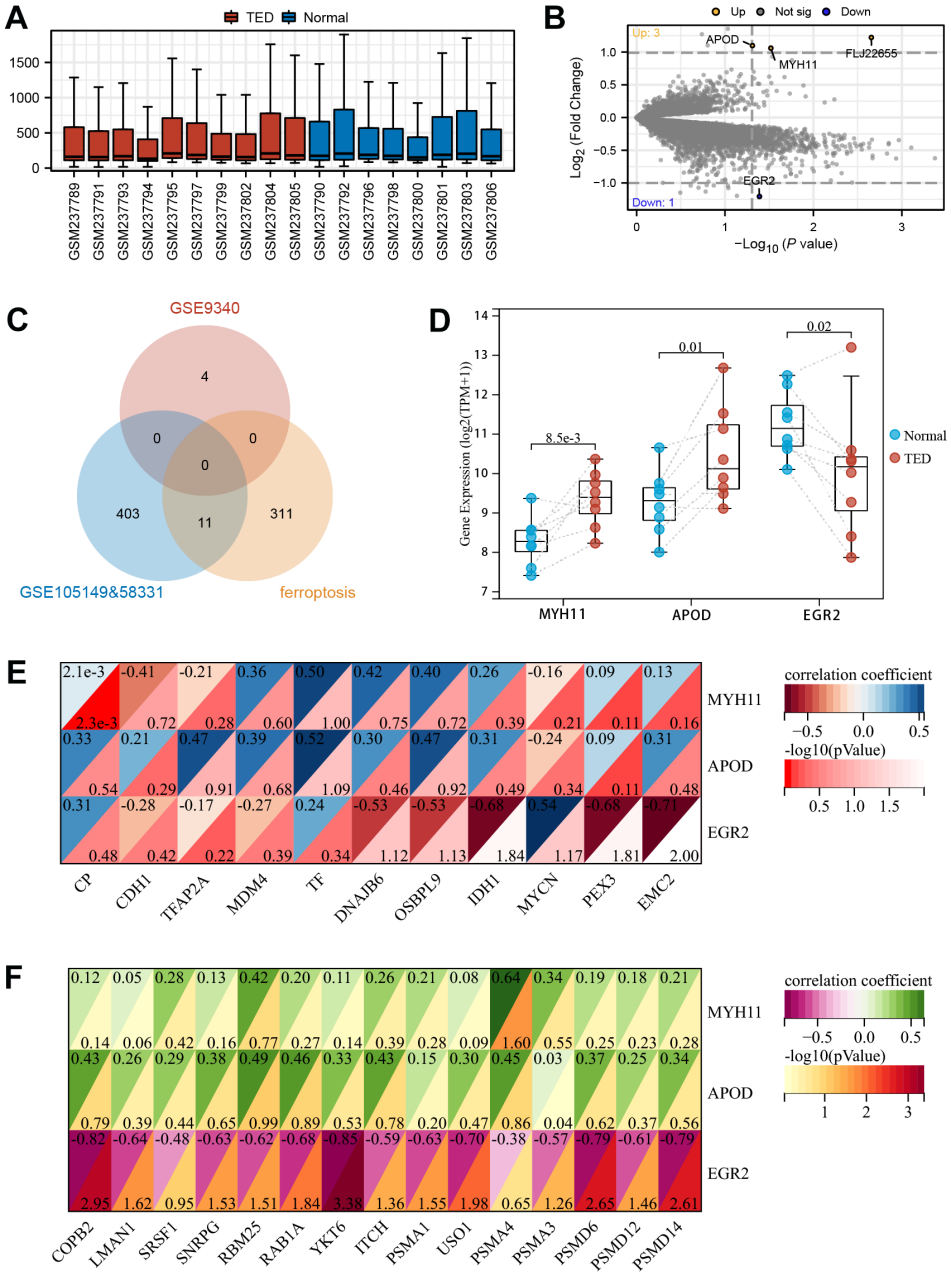
**Screening of DEGs in thyroid.** (**A**) Box plot of gene expression in TED-related datasets; (**B**) Screening of DEGs in thyroid samples; (**C**) Venn diagram of interaction among TED-related datasets and ferroptosis-related genes; (**D**) DEG expression levels between TED and normal groups; (**E**) Correlation analysis between DEGs in thyroid and F-DEGs; (**F**) Correlation analysis between DEGs in thyroid and hub genes. *P < 0.05, **P < 0.01, ***P < 0.001.

By following the aforementioned data processing procedure, we obtained standardized thyroid tissue data from the GSE9340 dataset. This dataset consisted of samples from 10 patients with TED and 8 samples from healthy controls (Figure 10A). DEGs were identified and chosen for further analysis based on the preestablished threshold (Figure 10B). Upon conducting an interaction analysis, we discovered that the genes identified in GSE9340, GSE105149, and 58331 and the ferroptosis gene set did not have any overlapping genes (Figure 10C). Overexpression of MYH11 (*p* = 8.5e-3) and APOD (*p* = 0.01) was observed in the TED group, while downregulated expression of EGR2 (*p* = 0.02) was observed in the TED group (Figure 10D).

We identified these three DEGs as T-DEGs and explored whether they were potentially related to F-DEGs and H-DEGs. The results showed that EGR2 was significantly negatively correlated with IDH1 (r = -0.68), PEX3 (r = -0.68), and EMC2 (r = -0.71) (all *p* < 0.05) (Figure 10E). Turning to H-DEGs, we observed a significant positive correlation between MYH11 and PSMA4 (r = 0.64, *p* < 0.05). In addition, EGR2 was negatively correlated with all H-DEGs, especially COPB2 (r = -0.82, *p* < 0.01), YKT6 (r = -0.85, *p* < 0.001), PSMD6 (r = -0.79, *p* < 0.01), and PSMD14 (r = -0.79, *p* < 0.01) (Figure 10F).

### Validation of the DEGs and immune infiltration

**Figure 11:**
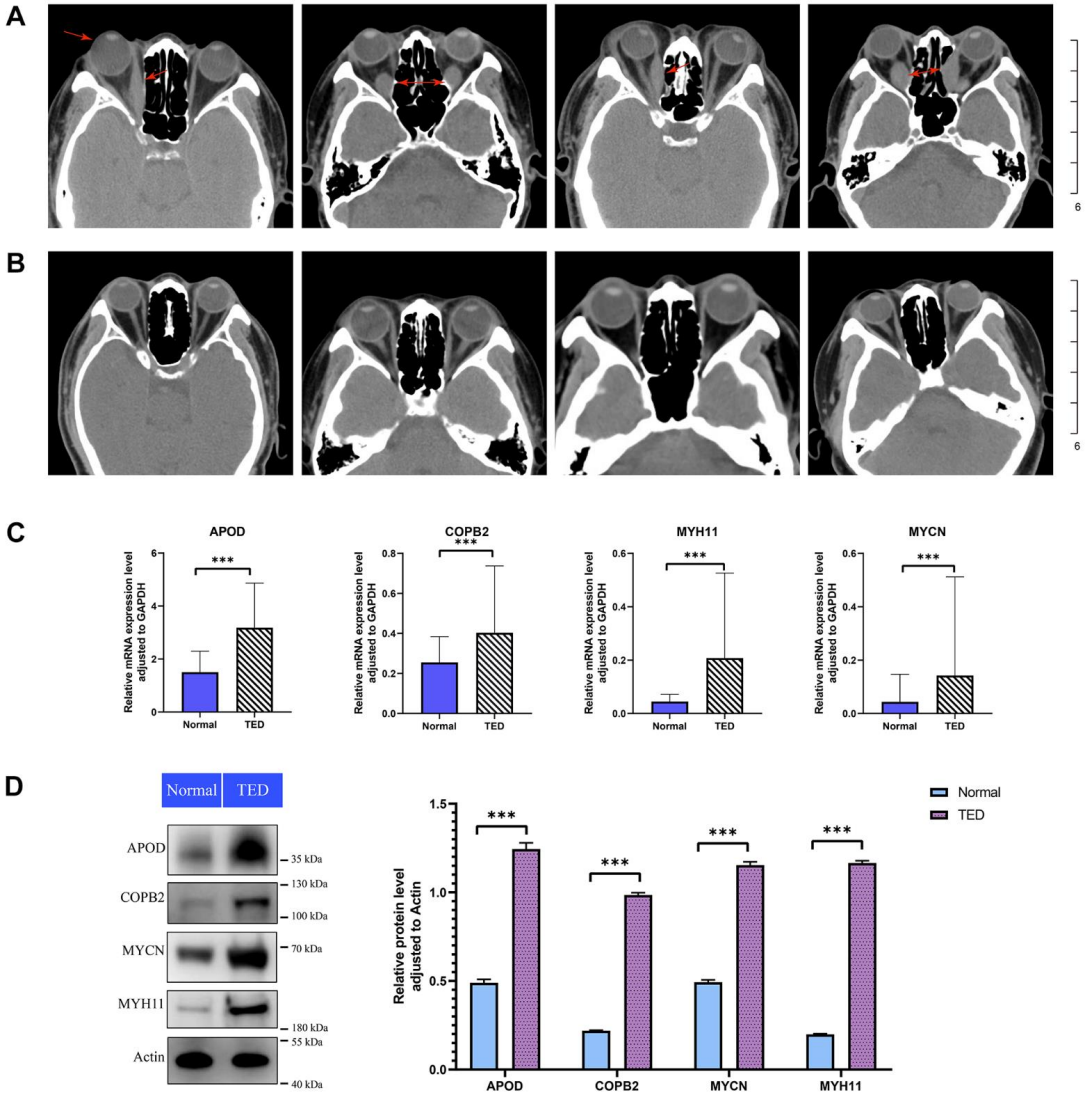
**Imaging analysis and experimental validation.** (**A**) CT images of patients with TED; red arrow: thickened rectus oculi and protruding bulbus oculi; (**B**) CT images of normal people; (**C**) qPCR for expression validation of the DEGs between TED and normal groups; (**D**) WB for expression validation of the DEGs between TED and normal groups. qPCR, quantitative polymerase chain reaction; WB, western blot. *P < 0.05, **P < 0.01, ***P < 0.001.

According to the computerized tomography (CT) scan, we could visually compare the degree of adipose infiltration and ocular muscle hypertrophy between the TED and normal groups. Patients with TED had obvious eye muscle hypertrophy and ocular exophthalmia (Figure 11A) compared with normal people (Figure 11B).

We further examined the expression of F-DEGs, H-DEGs, and T-DEGs in the periorbital adipose tissue. A total of 23 pairs of samples from patients with TED and normal controls were collected for qPCR validation. The expression of APOD, COPB2, MYH11, and MYCN in TED tissues was higher than that in normal tissues (all *p* < 0.001), as supported by qPCR (Figure 11C). Consistent with this result, WB further corroborated the higher expression of these four genes in TED than in normal tissues, with a significant difference (all *p* < 0.001) (Figure 11D).

**Figure 12:**
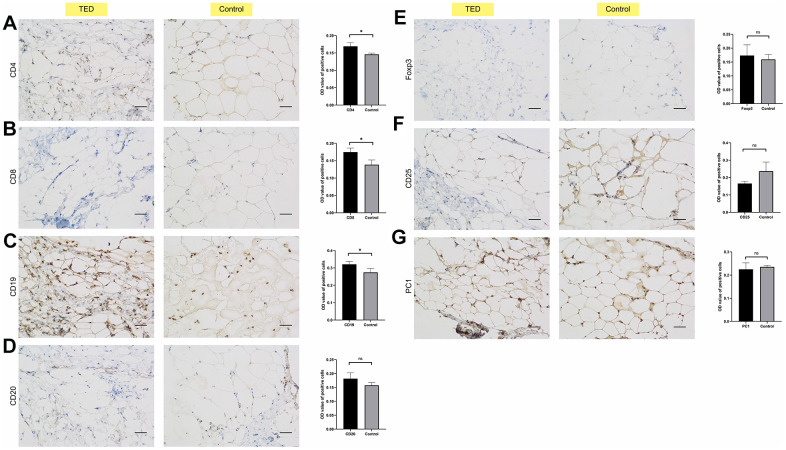
**Immunohistochemical validation of immune cell infiltration.** Identifying the differences in immune cell biomarkers and their presence in periorbital adipose tissue of thyroid ophthalmopathy patients and normal controls (x200). (**A**) CD4; (**B**) CD8; (**C**) CD19; (**D**) CD 20; (**E**) Foxp3; (**F**) CD25; (**G**) PC1. *P < 0.05, **P < 0.01, ***P < 0.001.

We also employed IHC to investigate the infiltration of immune cells in periorbital adipose tissue. According to the degree of staining in the cytoplasm and nuclei, we observed obviously upregulated expression of CD4, CD8, and CD19 in TED compared with normal tissues (all *p* < 0.05) (Figure 12A–12C). However, no significant differences in the expression of CD20, Foxp3, CD25, and PC1 were observed between the TED and control groups (all *p* > 0.05) (Figure 12D–12G).

## DISCUSSION

TED, as a common disease closely related to autoimmune thyroid disease, seriously affects the physical and mental health of patients [54]. In severe cases, people can even become disabled due to compression optic neuropathy or corneal decompensation [55, 56]. The pathogenesis of TED involves the deposition of hyaluronic acid and de novo synthesis of fat, resulting in orbital tissue expansion, muscle hypertrophy, and orbital inflammation caused by infiltration of various immune cells. Unfortunately, there is no cure for TED. Existing treatments, including high-dose glucocorticoid shock and orbital radiotherapy, have limited ability to alleviate the inflammatory reaction in the acute stage of the condition and are unable to rectify the long-term sequelae of the illness [57]. With the deepening of our understanding of the molecular pathways involved in the development of TED, targeted therapy is expected to become a new method for the treatment of TED. However, except for teprotumumab [58], there has been no breakthrough in this area of research. Currently, numerous studies have provided extensive evidence indicating that ferroptosis not only serves as a crucial factor in the onset and advancement of various cancers [59–62] but also actively contributes to the development, progression, and prognosis of several major chronic diseases [63–65]. This regulated cell death process, driven by lethal lipid peroxidation, participates in various cellular metabolic processes and disease-related signaling pathways, playing an essential role in tumor suppression, immune surveillance, and ischemia-reperfusion injury, among others [66]. Despite this, the mechanism of ferroptosis in TED remains a mystery. The accompanying study may reveal a possible link between ferroptosis and the emergence of TED, along with novel diagnostic indicators or therapeutic targets to address this gap. In the current work, we discovered ferroptosis-related DEGs in TED patients and built corresponding diagnostic prediction models through deep machine learning for the first time. Subsequently, we performed enrichment analyses on F-DEGs using GO/KEGG, GSEA, and GSVA, which indicated that the relevant molecular mechanisms were mainly focused on protein processing and transportation and hereditary material processing. Additionally, we employed WGCNA for modularization analysis of DEGs and obtained H-DEGs, which were completely different from F-DEGs. A PPI network was established, and the excellent diagnostic performance of H-DEGs was verified. Through immune infiltration analysis, special immune cells infiltrated in TED patients were identified, as were their potential correlations with the specific DEGs. Moreover, we conducted correlation analysis between T-DEGs, F-DEGs, and H-DEGs and found that EGR2 was highly negatively correlated with several DEGs in lacrimal gland samples. Adipose tissues from TED patients were also retrieved for further exploration and validation of DEG expression and immune cell infiltration.

In contrast to a previous bioinformatic study [22], our research integrated two TED-related datasets with batch effect removal to screen out brand-new DEGs. For the first time, 11 ferroptosis-related DEGs were identified as being involved in the occurrence and advancement of TED. After analyzing the expression level of every F-DEG, it was observed that all of them exhibited reduced expression in the TED group compared to the normal group, except MYCN (Figure 3A). We also revealed for the first time that these genes had an above-average diagnostic performance for TED (Figure 3B). While univariate analysis suggested that MYCN may be an effective diagnostic factor for TED (OR = 14.831, *p* = 0.005) (Figure 3C), the results of the multivariate analysis did not provide further validation. It is necessary to further validate its clinical value by expanding the sample size. Additionally, using machine learning, we constructed two pioneering ferroptosis-related diagnostic prediction models. Both of them were certified to be highly accurate and robust, with outstanding performance on clinical decisions (Figure 4). However, more clinical data should be incorporated for further validation of the reliability and precision of these models. Due to the lack of relevant data on prognosis and treatment, this study did not delve deeply into the clinical application of ferroptosis in TED therapy. However, we have planned to collaborate with more clinical centers and establish animal models to facilitate the feasibility of clinical translation practices.

According to reported studies, MYCN mutations have been strongly linked to neuroblastoma by upregulating the expression of the iron import transferrin receptor and targeting the Xc- system/glutathione (GSH) pathway [67, 68]. CDH1 is associated with colorectal cancer through its involvement in the E-cadherin-NF2-Hippo-YAP signaling pathway [66, 69]. MDM4 is implicated in breast cancer by negatively regulating p53 and influencing the stress response [66, 70]. Additionally, IDH1 is involved in cholangiocarcinoma by inducing the GPX4-regulated ferroptosis pathway [66, 71, 72]. In addition to these well-established classic ferroptosis pathways, our study identified an innovative finding that F-DEGs were involved in molecular pathways of protein processing and transportation. This discovery suggests a potential interaction between the ferroptosis mechanism and protein processing in TED patients. Based on the regulated nature of the iron death mechanism, we propose that all cellular activities, including cell proliferation, metabolism, and material transportation, are subject to its regulation. However, the precise regulatory relationship, participating pathways, and core targets require further experimental validation. Our results, which are consistent with prior research, also imply an essential role of intracellular protein processing in TED progression. Our study is the first to propose the involvement of ribosome-related mechanisms and ATP synthesis coupled electron transport pathway in TED, with potential shared mechanisms with neurodegenerative diseases such as Alzheimer’s disease.

Although some PSMD family genes were reported to be correlated with TED [22], we discovered a more comprehensive TED-related hub gene set (including six PSMD family genes) through WGCNA. Each of the H-DEGs’ diagnostic values was evaluated, and the ROC curves demonstrated their excellent performance for the first time (Figure 8H).

According to our knowledge, this study is the initial examination of the infiltration of immune cells in TED patients’ lacrimal gland tissues. According to the CIBERSORT algorithm, the TED group had a higher infiltration level of B cells, CD8 T cells, and Tregs but a lower infiltration level of plasma cells and CD4 T cells (Figure 9B). Positive correlations between M1 macrophages and naive B cells, activated mast cells and resting NK cells, and resting mast cells and M2 macrophages, as well as a negative correlation between resting memory CD4 T cells and plasma cells, were observed (Figure 9C). Potential correlations between F-DEGs and H-DEGs and immune cells were also analysed. Our analysis revealed a significant negative correlation between CDH1 and resting mast cells (*p* < 0.001), while a significant positive correlation was observed between CDH1 and CD8 T cells (*p* < 0.01). Additionally, we found a significant positive correlation between MDM4 and NK cells resting (*p* < 0.01) (Figure 9E). Although previous studies have suggested that the immune system may participate in tumor or inflammatory processes through certain key nodes of the ferroptosis pathway, the exact mechanisms remain unclear. Importantly, our study presents novel findings, as we are the first to propose the involvement of the immune system in the pathogenesis of autoimmune disease (TED) through potential nodes of the ferroptosis pathway. Hence, it is necessary to enlarge the sample for further validation and in-depth mechanism mining.

Moreover, we innovatively assessed the potential correlations between the DEGs from two different target organs of TED patients (thyroid and lacrimal glands). EGR2, which originated from the thyroid sample, was considered to be significantly negatively correlated with several DEGs from the lacrimal gland sample. In further basic experiments, we obtained consistent expression results for four DEGs (APOD, COPB2, MYH11, and MYCN) in the periorbital adipose tissue of TED patients by qPCR and WB. According to the IHC results, we speculated that CD4 T cells, CD8 T cells, and B cells infiltrated more in the periorbital adipose tissue of TED patients than in those of normal people.

This is the first study to include the three TED effector organs for a comprehensive multidimensional interaction analysis. In our seminal work, we used more scientific data processing methods to uncover new biomarkers and potential therapeutic targets. Previous studies [18, 22, 73] only focused on DEG screening in a single type of tissue. The results of their analyses were superficial and unconvincing to some extent due to a lack of external validation. The biggest highlight of our research is that diversified analytical and validation methods were employed to connect the internal links of the three target organs and revealed four novel DEGs co-expressed in three different tissues of TED patients.

Unfortunately, the study did not include the three target organ samples from the same individual, which somewhat reduced the validity and homogeneity of the study. The main reason for this dilemma was the difficulty of human sample acquisition. We envision improving the reliability and homogeneity of our findings by constructing a TED animal model in the future. Another main weakness of this study was the paucity of the sample size. Due to the niche nature of TED, it is difficult to obtain enough clinical samples in the short term. In the future, we can further incorporate more clinical centers or build animal models to expand the sample size. Third, the study did not further explore and verify the mechanisms and pathways between target organs, novel DEGs, and immune cells due to expense limitation. The inclusion of an external validation cohort and conducting multiple functional experiments are crucial for our research. The former will enhance the robustness and generalizability of the findings, while the latter will provide a deeper understanding of the unknown molecular mechanisms. We propose multidisciplinary collaboration and further fundamental experiments in the future to fill the gaps in this field. Despite its limitations, this study certainly adds to our understanding of TED pathogenesis. This work offers valuable insights for exploring novel targets and immune infiltration in TED.

The present study was conducted to extensively search for more valuable biomarkers and comprehensively evaluate the state of immune infiltration in TED to uncover new therapeutic approaches. The most obvious finding to emerge from this study was the discovery of previously unidentified biomarkers, namely, F-DEGs, H-DEGs, and T-DEGs, and the validation of the co-expression of APOD, COPB2, MYH11, and MYCN in three distinct target organs. Additionally, the study also revealed a strong correlation between TED pathogenesis and several types of immune cells, including CD4 T cells, B cells, and CD8 T cells. Collectively, these findings indicate that ferroptosis, along with immune injury induced by T lymphocytes and B lymphocytes, could potentially be the pivotal mechanisms implicated in TED pathogenesis. This work contributes to broadening the understanding of the molecular mechanisms underlying TED and suggests two primary areas of investigation, namely, ferroptosis and immune infiltration, as prospective focal points for further studies. The results of this research provide an experimental foundation for future investigation into the potential connection between ferroptosis, immune infiltration, and TED through a network pathway. Additionally, it illuminates the progress of novel treatment objectives for TED.

## Supplementary Material

Supplementary Table 1

Supplementary Tables 2 and 3
