# Supplementary material for: Novel insights into the pathogenesis of thyroid eye disease through ferroptosis-related gene signature and immune infiltration analysis
Source: Aging (Albany NY). 2024 Mar 25;16(7):6008–34. doi: 10.18632/aging.205685 (PMC11042930; doi:10.18632/aging.205685)
Supplement: Supplementary Table 1 [file aging-16-205685-s001.docx]

**Supplementary Table 1. Baseline characteristics of 23 samples in TED group (1).**

| **No.** | **ID** | **Gender** | **Age** | **Eye** | **Orbital decompression** | **Eyelid surgery** | **Strabismus operation** | **diplopia** | **Corneal lesion** | **Eyelid retraction** | **TPOAb** | **TgAb** | **TRAb** |
| --- | --- | --- | --- | --- | --- | --- | --- | --- | --- | --- | --- | --- | --- |
| 1 | 21055558 | F | 33 | OD | ✓ |  |  |  |  |  | 992 | 19.9 | 0.25 |
|  | 21042834 |  |  | OS | ✓ |  |  |  |  |  |  |  |  |
| 2 | 21019639 | M | 39 | OS | ✓ |  |  | ✓ |  | ✓ | 134.8 | 18.9 | 2.78 |
| 3 | 21052949 | F | 48 | OD | ✓ |  |  | ✓ |  | ✓ | 64.3 | <15 | 2.31 |
|  | 21062474 |  |  | OS | ✓ |  |  |  |  |  |  |  |  |
|  | 21090623 |  |  | OU |  |  | ✓✓ |  |  |  |  |  |  |
| 4 | 21041857 | M | 53 | OS | ✓ |  |  | ✓ |  | ✓ | 307 | 259.4 | 1.76 |
|  |  |  |  | OD | ✓ |  |  |  |  |  |  |  |  |
|  | 21077190 |  |  | OU |  |  | ✓✓ |  |  |  |  |  |  |
| 5 | 21051777 | M | 41 | OD | ✓ |  |  | ✓ |  | ✓ | 278 | 1647.1 | 4.84 |
|  |  |  |  | OD |  | ✓ |  |  |  |  |  |  |  |
|  | 21070962 |  |  | OD |  |  | ✓✓ |  |  |  |  |  |  |
|  |  |  |  | OD |  | ✓ |  |  | ✓ |  |  |  |  |
| 6 | 21058376 | M | 32 | OD | ✓ |  |  | ✓ |  | ✓ | 0.2 | 0.6 | 10.2 |
|  |  |  |  | OS | ✓ |  |  |  |  |  |  |  |  |
|  | 21071086 |  |  | OU |  | ✓✓ |  |  | ✓ |  |  |  |  |
| 7 | 21057417 | F | 26 | OS | ✓ |  |  |  |  |  | <15 | <15 | 1.2 |
|  |  |  |  | OD | ✓ |  |  |  |  |  |  |  |  |
| 8 | 21030823 | F | 52 | OD | ✓ |  |  |  |  | ✓ | 29.6 | 9.1 | 1.58 |
|  | 21062536 |  |  | OD |  |  | ✓ |  |  |  |  |  |  |
| 9 | 21045647 | M | 44 | OS | ✓ |  |  |  |  |  | 318.9 | 61.4 | 1.5 |
|  | 21063647 |  |  | OD | ✓ | ✓✓ |  |  |  |  |  |  |  |
| 10 | 21066069 | F | 34 | OD | ✓ |  |  |  |  | ✓ | <15 | <15 | 0.82 |
|  |  |  |  | OS | ✓ | ✓✓ |  |  | ✓ |  |  |  |  |
|  |  |  |  | OD |  | ✓✓ |  |  |  |  |  |  |  |
| 11 | 21049904 | F | 50 | OS | ✓ |  |  | ✓ |  | ✓ | 1273.8 | <15 | 20.5 |
|  | 21069999 |  |  | OD | ✓ | ✓ |  |  |  |  |  |  |  |
|  | 21102431 |  |  | OD |  |  | ✓ |  |  |  |  |  |  |
| 12 | 21071986 | F | 32 | OU | ✓✓ |  |  |  |  | ✓ | 3.6 | 2.5 | 1.12 |
|  | 21095223 |  |  | OS |  | ✓ |  |  |  |  |  |  |  |
| 13 | 21018791 | F | 51 | OU | ✓✓ |  |  | ✓ |  | ✓ | 28 | 15 | 24.61 |
|  | 21033367 |  |  | OU |  | ✓✓ |  |  |  |  |  |  |  |
|  | 21044767 |  |  | OU |  |  | ✓✓ |  |  |  |  |  |  |
| 14 | 21046985 | M | 26 | OD | ✓ |  |  | ✓ |  | ✓ | <28 | <15 | 0.24 |
| 15 | 21085472 | F | 60 | OS | ✓ |  |  | ✓ |  |  | <28 | <15 | 4.51 |
|  | 21098307 |  |  | OS |  |  | ✓ |  |  |  |  |  |  |
| 16 | 21027953 | M | 58 | OU | ✓✓ |  |  |  | ✓ | ✓ | <28 | <15 | 0.73 |
|  |  |  |  |  |  |  |  |  | ✓ |  |  |  |  |
| 17 | 21066889 | M | 44 | OU | ✓✓ |  |  | ✓ |  |  | <28 | <15 | 0.25 |
|  |  |  |  |  |  |  |  |  |  |  |  |  |  |
| 18 | 21021671 | F | 48 | OD | ✓ |  |  | ✓ |  | ✓ | 0.5 | 0.8 | 0.21 |
|  |  |  |  | OD |  | ✓ |  |  |  |  |  |  |  |
| 19 | 21104347 | F | 28 | OD | ✓ |  |  |  | ✓ | ✓ | <15 | <15 | 0.28 |
| 20 | 21075290 | M | 53 | OD | ✓ |  |  | ✓ |  | ✓ | 12.3 | 28.9 | 1.67 |
|  |  |  |  | OS | ✓ |  |  |  |  |  |  |  |  |
|  | 22006217 |  |  | OU |  |  | ✓ |  |  |  |  |  |  |
| 21 | 21083192 | M | 48 | OS | ✓ |  |  | ✓ |  | ✓ | 46.2 | <15 | 0.29 |
|  |  |  |  | OS |  | ✓ | ✓ |  | ✓ |  |  |  |  |
|  | 21096001 |  |  | OD | ✓ |  |  |  |  |  |  |  |  |
|  |  |  |  | OU |  | ✓✓ | ✓✓ |  |  |  |  |  |  |
|  |  |  |  | OU |  |  |  |  |  |  |  |  |  |
| 22 | 21099164 | F | 44 | OD | ✓ |  |  |  |  | ✓ | 36.7 | 15.7 | >30 |
|  | 22011520 |  |  | OS | ✓ |  |  |  |  |  |  |  |  |
| 23 | 21075422 | M | 52 | OD | ✓ |  | ✓ | ✓ |  | ✓ | 38.6 | <15 | 1.39 |

F, female; M, male; OD, Oculus Dexter; OS, Oculus Sinister; OU, Oculus Uterque; EP, eyeball protrusion; IOP, intraocular pressure.

✓✓ means binoculus.

**Supplementary Table 1. Baseline characteristics of 23 samples in TED group (2).**

| No. | ID | Vision | | IOP | | EP | | CT | |
| --- | --- | --- | --- | --- | --- | --- | --- | --- | --- |
|  |  | Preoperative | Postoperative | Preoperative | Postoperative | Preoperative | Postoperative | Muscle expansion | Fat hyperplasia |
| 1 | 21055558 | 0.6/0.8 | 0.6/0.8 | 16/20 | 20/18 | 18-108-18 | 16.5-108-18 |  | ✓ |
|  | 21042834 | 1.0x-0.75DS | 1.0x-0.75DS | 23.7/25 | 19.7/18 | 15.5-103-18 | 15.5-103-16 |  | ✓ |
| 2 | 21019639 | 1.5/1.5 | 1.5/1.5 | 23/23.3 | 19/19 | 17-107-19 | 17-107-17 | Inferior rectus |  |
| 3 | 21052949 | 1.0/0.8 | 0.8/0.8 | 27/25 | 29.7/31.7 | 19.5-100-18 | 17.5-102-18 | Inferior internal and external rectus |  |
|  | 21062474 | 1.0/0.8 | 1.0/1.0 | 26/29.3 | 23/20 | 16.5-102-20 | 17-102-17 | Inferior internal and external rectus |  |
|  | 21090623 |  |  |  |  |  |  |  |  |
| 4 | 21041857 | 0.8/0.8 | 1.0/1.0 | 23/23 | 16/15 | 19-105-21 | 16-102-15.5 | Superior Inferior and Medial rectus |  |
|  |  |  |  |  |  |  |  | Superior Inferior and Medial rectus |  |
|  | 21077190 |  |  |  |  |  |  |  |  |
| 5 | 21051777 | 0.2/0.5 | 0.2/0.5 | 24/11 | 12/11 | 23-113-20 | 20-116-21 | Superior Inferior and Medial rectus |  |
|  |  |  |  |  |  |  |  |  |  |
|  | 21070962 | 0.2/0.5 |  | 24/11 | 25/15 | 26-113-21 | 20-116-21 |  |  |
|  |  |  |  |  |  |  |  |  |  |
| 6 | 21058376 | 1.0/1.0 | 1.0/1.0 | 21/23 | 12/13 | 26.5-115-26.5 | 23-114-23 | Inferior rectus |  |
|  |  |  |  |  |  |  |  | Inferior rectus |  |
|  | 21071086 |  |  |  |  |  |  |  |  |
| 7 | 21057417 | 1.0/1.0 |  | 14/14 |  | 19-107-19 | 14-107-13 |  | ✓ |
|  |  |  |  |  |  |  |  |  | ✓ |
| 8 | 21030823 | 1.0/1.0 | 18/18 | 1.0/1.0 | 19/19 | 19-101-15 | 15-101-15 | Inferior and Medial rectus |  |
|  | 21062536 |  |  |  |  |  |  |  |  |
| 9 | 21045647 | 0.5/0.15 | 0.5/0.15 | 19.3/15 | 13/12.3 | 26-115-26 | 26-115-24 |  | ✓ |
|  | 21063647 | 0.5/0.12 |  | 16/12 |  | 28-115-25 | 24-115-24 |  | ✓ |
| 10 | 21066069 |  |  |  |  |  |  | Superior Inferior and Medial rectus |  |
|  |  | 1.0/0.7 | 0.8/0.8 | 18/17 | 17/17 | 17-105-16 | 13-105-13 | Superior and Inferior rectus |  |
|  |  |  |  |  |  |  |  |  |  |
| 11 | 21049904 | 0.8/1.0 |  | 20/18 |  | 20-97-21 | 20-100-17 | Superior Inferior and Medial rectus |  |
|  | 21069999 | 0.8/1.0 |  | 20/18 | 28/25 | 19-98-16 | 17-98-16 | Superior Inferior and Medial rectus |  |
|  | 21102431 |  |  |  |  |  |  |  |  |
| 12 | 21071986 | 1.2/1.2 |  | 23/23 | 21/20 | 23-105-23 | 20-105-19 |  | ✓ |
|  | 21095223 |  |  |  |  | 18-106-18 |  |  | ✓ |
| 13 | 21018791 | 0.4/0.6 | 0.8/1.0 | 22/20.5 | 20.8/22.3 | 25-109-24 | 22-109-21 | Superior Inferior and Medial rectus |  |
|  | 21033367 |  |  |  |  |  |  | Superior Inferior and Medial rectus |  |
|  | 21044767 | 0.8/1.0 |  | 21/19 |  |  |  |  |  |
| 14 | 21046985 | 0.8/0.8 |  | 12/13 |  | 18-107-18 | 16-105-17 |  | ✓ |
| 15 | 21085472 | 1.0/0.6 | 1.0/0.8 | 22/32 | 16/16.5 | 15-97-18 | 16-96-15 | Inferior and Medial rectus |  |
|  | 21098307 |  |  |  |  |  |  |  |  |
| 16 | 21027953 | 0.5/0.6 | 0.8/0.8 | 12/20 | 12/12 | 23-107-21 | 17-100-16 | Superior and Inferior rectus |  |
|  |  |  |  |  |  |  |  | Superior Inferior and Medial rectus |  |
| 17 | 21066889 | 1.2/1.5 | 1.2/1.5 | 11/12.5 | 14/14 | 22-116-21.5 | 18-116-17 |  | ✓ |
|  |  |  |  |  |  |  |  |  | ✓ |
| 18 | 21021671 | 0.8/1.0 |  | 17/21.5 |  | 18-104-16.5 |  | Superior rectus |  |
|  |  |  |  |  |  |  |  |  |  |
| 19 | 21104347 | 1.0/1.0 | 1.0/1.0 | 21.7/20 | 22/17 | 24-105-21 | 20-105-21 | Superior rectus |  |
| 20 | 21075290 | 1.0/1.0 | 1.0/1.0 | 14/18 |  | 23-112-22 | 21-105-21 | Superior Inferior and Medial rectus |  |
|  |  |  |  |  |  |  |  | Superior and Inferior rectus |  |
|  | 22006217 |  |  |  |  |  |  |  |  |
| 21 | 21083192 | 1.0/0.8 | 1.0/0.8 | 27/30 | 15.7/19.8 | 23-100-23 | 23-100-20.5 | 4 rectus |  |
|  |  |  |  |  |  |  |  |  |  |
|  | 21096001 | 1.0/0.8 | 1.0/0.8 | 25/35 | 16.4/18.9 | 23-100-20 | 20-100-19 | 4 rectus |  |
|  |  |  |  |  |  |  |  |  |  |
|  |  |  |  |  |  |  |  |  |  |
| 22 | 21099164 | 1.5/1.5 |  | 17/18 |  | 23-101-20 | 17-100-21 | Superior Inferior and Medial rectus |  |
|  | 22011520 |  |  |  |  |  |  | Superior Inferior and Medial rectus |  |
| 23 | 21075422 | 1.2/1.5 | 1.2/1.5 | 17/11.7 | 13/14 | 18.5-100-15 | 15-100-15 | Superior Inferior and Medial rectus |  |

F, female; M, male; OD, Oculus Dexter; OS, Oculus Sinister; OU, Oculus Uterque; EP, eyeball protrusion; IOP, intraocular pressure.

✓✓ means binoculus.
