# Supplementary material for: Novel insights into the pathogenesis of thyroid eye disease through ferroptosis-related gene signature and immune infiltration analysis
Source: Aging (Albany NY). 2024 Mar 25;16(7):6008–34. doi: 10.18632/aging.205685 (PMC11042930; doi:10.18632/aging.205685)
Supplement: Supplementary Tables 2 and 3 [file aging-16-205685-s002.pdf]

Supplementary Table 2. Baseline characteristics of 23 samples in normal group.

No.	ID	Gender	Age	Surgery	Eye	EP	CT
1	21004076	F	56	Orbital fracture repair	OD	11-112-14	Fracture of medial orbital wall
2	21002345	M	65	Orbital fracture repair	OD	12-105-16	Fracture of inferior orbital wall
3	21003038	F	36	Orbital fracture repair	OS	14-104-12	Fracture of medial orbital wall
4	20049698	F	36	blepharoplasty	OD	/	/
5	21005477	F	40	Orbital fracture repair	OD	14-106-16	Fracture of inferior orbital wall
6	21003596	M	37	Orbital fracture repair	OS	16-106-14	Fracture of medial orbital wall
7	21000884	F	43	blepharoplasty	OS	/	/
8	21004349	M	41	Orbital fracture repair	OS	15-110-13	Fracture of medial orbital wall
9	21002403	F	52	blepharoplasty	OD	/	/
10	21008886	F	32	Orbital fracture repair	OD	14-108-16	Fracture of medial orbital wall
11	20050052	F	23	blepharoplasty	OS	/	/
12	21006940	M	28	Orbital fracture repair	OS	15-104-12	Fracture of medial orbital wall
13	21008307	M	77	Orbital fracture repair	OD	13-110-16	Fracture of medial orbital wall
14	21008395	M	33	Orbital fracture repair	OD	17-120-19	Fracture of inferior orbital wall
15	20051723	M	60	Orbital fracture repair	OS	15-109-12	Fracture of inferior orbital wall
16	21022414	M	37	Orbital fracture repair	OD	15-118-18	Fracture of inferior orbital wall
17	21018808	M	50	Orbital fracture repair	OS	20-115-19	Fracture of medial orbital wall
18	21023630	M	52	Orbital fracture repair	OD	18-117-16	Fracture of inferior orbital wall
19	21028737	F	58	blepharoplasty	OS	/	/
20	21029104	F	65	blepharoplasty	OD	/	/
21	21042246	F	56	blepharoplasty	OD	/	/
22	21014687	F	58	blepharoplasty	OS	/	/
23	21020717	F	52	blepharoplasty	OS	/	/

F, female; M, male; OD, Oculus Dexter; OS, Oculus Sinister; OU, Oculus Uterque; EP, eyeball protrusion.

Supplementary Table 3. Basic information of primary antibodies for WB and IHC.

	No.	Target	Company	Product code	Species
WB	1	APOD-33KD	Proteintech	10520-1-AP	Rabbit
	2	COPB2-102KD	ABclonal	A21294	Rabbit
	3	MYCN-50KD	Proteintech	10159-2-AP	Rabbit
	4	MYH11-200KD	Proteintech	21404-1-AP	Rabbit
IHC	1	CD8 Monoclonal antibody	Proteintech	66868-1-Ig	Mouse
	2	CD4 Monoclonal antibody	Proteintech	67786-1-Ig	Mouse
	3	CD19 Monoclonal antibody	Proteintech	66298-1-Ig	Mouse
	4	CD20 Monoclonal antibody	Proteintech	60271-1-Ig	Mouse
	5	Anti-FOXP3 [236A/E7]	Abcam	Ab20034	Mouse
	6	IL-2RA/CD25 antibody	Affinity	AF7675	Rabbit
	7	PC1 antibody	Affinity	DF14432	Rabbit

WB, western blot; IHC, immunohistochemistry.
